# Molecular Identification and Dual Functions of Two Different CXC Chemokines in Nile Tilapia (*Oreochromis niloticus*) against *Streptococcus agalactiae* and *Flavobacterium columnare*

**DOI:** 10.3390/microorganisms8071058

**Published:** 2020-07-16

**Authors:** Chatsirin Nakharuthai, Prapansak Srisapoome

**Affiliations:** 1 Laboratory of Aquatic Animal Health Management, Department of Aquaculture, Faculty of Fisheries, Kasetsart University, 50 Paholayothin Rd, Ladyao, Chatuchak, Bangkok 10900, Thailand; chatsirin_nak@sut.ac.th; 2 Center of Advanced Studies for Agriculture and Food, Kasetsart University Institute for Advanced Studies, Kasetsart University (CASAF, NRU-KU), Bangkok 10900, Thailand; 3 School of Animal Technology and Innovation, Institute of Agricultural Technology, Suranaree University of Technology, 111 University Avenue, Muang, Nakhon Ratchasima 30000, Thailand

**Keywords:** Nile tilapia, immune responses, CXC chemokines, genes, recombinant proteins, biological function, phagocytosis, antibacterial activity

## Abstract

Two CXC chemokines in Nile tilapia (*On*-CXC1 and *On*-CXC2) were identified at both the genomic and proteomic levels. A southern blot analysis and comparison searching in Ensembl confirmed the typical structure of the CXC chemokine genes and provided evidence for unusual mechanisms used to generate the two different CXC chemokine transcripts that have not been reported in other vertebrate species so far. The expression levels of *On*-CXC1 and *On*-CXC2 were analyzed by quantitative real-time PCR. These two mRNAs were detected in various tissues of normal Nile tilapia, especially in the spleen, heart, and head kidney, indicating a homeostatic function in immunosurveillance. A time-course experiment clearly demonstrated that these two transcripts were effectively enhanced in the head kidney, spleen and trunk kidney of Nile tilapia 6, 12 and 24 h after injection with *Streptococcus agalactiae* but were down-regulated in all tested tissues at 48 h, reflecting the fact that they have short half-lives during the crucial response to pathogens that is characteristic of CXC chemokine genes in other vertebrates. Functional analyses obviously exhibited that these two CXC chemokines at concentrations of 1–10 μg strongly inactivated *S. agalactiae* and *Flavobacterium columnare* and effectively induced phagocytosis of leukocytes in vitro.

## 1. Introduction

Tilapia is one of the most popular fish cultured in global aquaculture after carp and salmonid fish. Since the late 1990s, the large-scale production and international trade of Nile tilapia has grown rapidly, and this fish has become an important commodity in international markets. The global production of farmed tilapia reached 4.6 and 6.5 million metric tons in 2013 and 2019, respectively [1]. In Thailand, aquaculture of Nile tilapia is the largest source of freshwater fish production and has expanded throughout the country since 2008. As Nile tilapia are tolerant of undesirable conditions, such as poor water quality, low dissolved oxygen levels and other stress conditions, compared with other commercial fish species, they are an ideal species for freshwater fish farming. Consequently, intensive production of Nile tilapia has been rapidly developed to increase their yield. However, this practice has led to more undesirable conditions than other systems, and it is the cause of a higher number of infectious agents, including parasites, viruses and bacteria [2]. Among these pathogens, *Streptococcus agalactiae* (Group B *Streptococcus*; GBS) is a common pathogenic bacterium that is a major cause of streptococcosis in Nile tilapia in Thailand. *S. agalactiae* is a beta-hemolytic, Gram-positive, non-motile, non-acid-fast and non-spore-forming bacterium. The clinical signs of S. agalactiae in Nile tilapia include septicemia, exophthalmia, corneal opacity, melanosis, swimming abnormalities, swelling and hemorrhage of the internal organs [3,4]. Death usually occurs over a short period, approximately 10 days [5]. Additionally, the Gram-negative, long rod and non-spore-forming bacterium *Flavobacterium columnare* was also observed to kill large numbers of Nile tilapia of various sizes. Thus, the diseases caused by these bacteria have a large impact on Nile tilapia cultures and are major problems for Nile tilapia farming in Thailand. Most farmers use a variety of chemicals and drugs to control this disease. However, the application of drugs and chemicals directly affects consumer health and the aquatic environment. To minimize drug usage, it is important to understand the fish immune system and consequently develop prophylactic or therapeutic strategies applicable to Nile tilapia aquaculture.

In all vertebrates, including fish, innate and adaptive immune responses protect against diseases [6]. Innate immunity is the first line of defense against pathogens and is the earliest and most basic immune mechanism [7]. This system comprises a variety of immune cells and mechanisms that defend the host from infection by pathogens. In the innate immune system, cytokines are a critical group of cell-signaling molecules that act as a bridge between the innate and adaptive immune systems [8]. They are produced and secreted by numerous cells that mediate and regulate the immune responses of vertebrates [9]. Chemokines are a superfamily of cytokines with chemoattractant properties that induce directed chemotaxis and attract a variety of leukocytes to sites of infection [10]. The chemokine family is divided into four major groups depending on the organization of their four characteristic cysteines and the spacing of their first two cysteine residues [11]. The four major groups of chemokines are CXC (α-chemokine), C (γ-chemokines), CX3C (δ-chemokines) and CC (β-chemokines) [12]. Of these, CXC chemokines belong to an important chemokine family that is directly involved in the pro-inflammatory process. This family contains a number of members belonging to the CXC chemokine subfamily, including CXCa, CXCb, CXCc, CXCd, CXCL12, CXCL14 and interleukin-8 (IL-8) in teleost fish [13]. They are known regulators of immune cell migration (e.g., neutrophils, basophils, and lymphocytes), and their expression can be induced by stimulating factors, such as pro-inflammatory cytokines, bacterial lipopolysaccharides, viral products or cellular stress [14]. Moreover, these molecules are released to signal to other immune cells to migrate to the site of inflammation. Structurally, CXC chemokines are small proteins, and the two N-terminal cysteines of CXC chemokines are separated by one non-conserved amino acid residue that is represented by an X (any) between two cysteine residues (CXC). Their target receptors are integral membrane proteins, including CXCR1/CXCR2, CXCR3, CXCR4, CXCR5, CXCR6 and CXCR7, which are G protein-coupled receptors. CXC chemokines are produced by a wide variety of cells, including macrophages, lymphocytes, epithelial cells and endothelial cells, in response to many different stimuli. The CXC chemokine subfamily can be divided into two subgroups based on the presence or absence of a glutamine-leucine-arginine (Glu-Leu-Arg; ELR) motif, which is associated with specificity for neutrophils. This motif precedes the first two cysteines in the NH2-terminal region [15]. Therefore, the primary function of the ELR-containing CXC chemokines (ELR+) is to recruit and activate neutrophils, T-lymphocytes and basophils but not monocytes and macrophages [16,17]. Moreover, they are important promoters of angiogenesis. Non-ELR-containing chemokines (ELR-) are believed to attract T-lymphocytes and NK cells and can inhibit endothelial cell proliferation, migration and angiogenesis [18]. Many of the CXC chemokines in higher vertebrates, including IL-8, are members of the subgroup containing the ELR+ motif. However, most fish CXC chemokines lack the ELR motif, although haddock (*Melanogrammus aeglefinus*) and Atlantic cod (*Gadus morhua*) [19] are exceptions. In addition, the ELR motif is sometimes replaced by a defective DLR motif (Asp-Leu-Arg) in trout [20]. Although trout lack the ELR motif preceding the CXC motif, they have a similar motif (DLR) in this position that retains the neutrophil-attracting ability of CXCL8 in humans [21]. Among the CXC chemokines, IL-8 was the first identified in mammals as a novel type of neutrophil-activating cytokine (CXCL8) [22]. In fish, the first reported CXC chemokine was isolated in 1999 from the lamprey (*Lampetra fluviatilis*), which is a jawless fish (agnathan) [23]. Interestingly, although it encodes a CXCL8-like peptide, the ELR motif associated with CXCL8 neutrophil-attracting ability in mammals and birds is absent from the lamprey IL-8. Following the discovery of a CXC chemokine in lamprey, this gene was cloned and sequenced in several teleost species, such as flounder [24]. The putative flounder IL-8 amino acid sequence showed 25% identity and 52% similarity to that of the lamprey, suggesting an evolutionary relationship between IL-8 molecules in jawed and jawless fish. The trout and bighead carp IL-8 genes, which have a DLR motif, were identified in 2002 [20,25]. Additionally, many different CXC chemokine genes have also been characterized in several fish species [16,19,26,27,28,29,30,31,32,33,34].

Antimicrobial peptides (AMPs) are effective components of the innate immune system for providing an essential first-line defense against a wide range of microorganisms (e.g., Gram-positive and -negative bacteria, fungi as well as viruses) [35,36]. Generally, AMPs possess positive charges at neutral pH (high p*I* values), which are attracted and incorporated into negatively charged bacterial membranes, causing membrane disruption and loss of intracellular constituents [37]. Over the years, several studies have reported that chemokines exhibit not only their main function as chemotactic cytokines but also antimicrobial activity with broad-spectrum function. Research to date has revealed that the structure of chemokines is highly conserved in a manner similar to that of members of the defensin class of AMPs. In teleosts, such as rainbow trout [38], catfish [39] and channel catfish [40], several chemokines also exert antimicrobial activity.

In the present study, two different CXC chemokines of the Nile tilapia were successfully cloned and characterized at both the genomic and proteomic levels. A southern blot analysis was employed to estimate the copy number of CXC chemokine genes in the Nile tilapia genome. The analysis of the genomic segment using the Ensembl Nile tilapia genomic databases (www.ensembl.org) and PCR was conducted to elucidate the full CXC gene organization in the Nile tilapia genome. The expression of these CXC chemokine mRNAs in various tissues of normal fish and in fish inoculated with *S. agalactiae* at different concentrations was analyzed using semi-quantitative RT-PCR and quantitative real-time PCR. Two recombinant CXC proteins were produced and tested for their biological functions regarding the chemotactic induction of phagocytosis and antimicrobial killing of pathogenic bacteria that caused severe disease outbreaks in Nile tilapia.

## 2. Materials and Methods

### 2.1. Experimental Animals

Healthy Nile tilapias (100 g) were obtained from Manit Genetics Co., Ltd., (Khao Yoi, Petchaburi Province, Thailand) and was acclimatized under laboratory conditions at the laboratory of Aquatic Animal Health Management, Department of Aquaculture, Faculty of Fisheries, Kasetsart University (Bangkok, Thailand). Subsequently, the experimental fish was maintained in an 80-L glass tank containing freshwater with an aeration system and fed twice a day with commercial feed at 5% body weight. The protocol was approved by the Animal Ethics Committee, Kasetsart University, Thailand (ethics ID: ACKU61-FIS-004).

### 2.2. Total RNA and mRNA Preparation

After acclimating for 7 days, the experimental fish was sacrificed, and the head kidney and spleen were collected. Total RNA was isolated from these two organs using TRIzol reagent (Gibco BRL, Carlsbad, CA, USA). Then, a QuickPrep Micro mRNA Purification kit (Amersham Biosciences, Buckinghamshire, UK) was used to isolate the mRNA following the manufacturer’s instructions, and the samples were stored at −80 °C until use.

### 2.3. 3′ and 5′ Ready-to-Use First-Strand cDNA Synthesis

In this experiment, 0.5 and 0.5 μg of mRNA from the spleen and head kidney, respectively, was pooled and used as a template to synthesize the 3′ and 5′ first-strand cDNAs using a SMARTer™ RACE cDNA amplification kit (Clontech, CA, USA) following the company’s protocol.

### 2.4. Rapid Amplification of cDNA Ends (RACE) PCR

Using the isolated mRNA, two uncharacterized cDNAs of the EST clones HK10 (FF279523) and HK737 (FF280148), which are homologous to the CXC chemokines of other vertebrate species, were obtained from the head kidney cDNA library of Nile tilapia [41]. These full-length cDNAs were named *On*-CXC1 and *On*-CXC2. Based on comparisons with published sequences using the BLAST programs from the GenBank database (http://www.ncbi.nlm.nih.gov), it was clearly demonstrated that EST clone HK10 contained the full sequence of *On*-CXC1 and clone HK737 possessed a partial nucleotide sequence of *On*-CXC2.

To obtain the additional information regarding the cDNA sequence of clone HK737, 3′ and 5′ RACE PCRs were performed using the 3′ and 5′ ready-to-use first-strand cDNA prepared in Section 2.3 with a SMARTer™ PCR cDNA synthesis kit (Clontech, USA) following the manufacturer’s instructions. The primers CXC-2F and CXC-2AR (Table S1) were designed based on the EST clone HK737 for 3′ and 5′ RACE PCR, respectively. The RACE PCR reaction (final volume of 25 µL) contained 1.25 µL 3′ or 5′ RACE-ready cDNAs, 2.5 μL TaKaRa LA *Taq* buffer, 0.5 μL 10 mM dNTP mix (2.5 mM each), 0.5 μL (2.5 unit) TaKaRa LA *Taq* DNA Polymerase, 2.5 μL 10× universal primer mix, 0.4 μM UPM-long, 2 μM UPM-short (Table S1), 1.0 μL 10 μM gene-specific primers (CXC-2F for 3′ RACE or CXC-2AR for 5′ RACE, Table S1) and 16.75 μL distilled water. Touchdown PCR was used for RACE amplification. The PCR steps were as follows: 94 °C for 30 s, 72 °C for 3 min for 5 cycles; 94 °C for 30 s, 68 °C for 30 s, 72 °C for 3 min for 5 cycles; 94 °C for 30 s, 55 °C for 30 s, 72 °C for 3 min for 25 cycles.

### 2.5. Cloning and Characterization of the Full-Length CXC Chemokine cDNA of Nile Tilapia

The PCR products from Section 2.4 were run on a 1.5% agarose gel, purified using a QIAquick^®^ gel extraction kit (Qiagen, Venlo, Netherlands), cloned into the pGEM-T Easy vector (Promega, Madison, WI, USA), and then ligated and transformed into Escherichia coli (strain JM109) following the manufacturer’s instructions. Then, plasmid was extracted from the positive clones using a Plasmid DNA Extraction Manual Kit (Bio Excellence, Thailand). Finally, nucleotide sequencing of the target plasmid in both the 5′ and 3′ directions was conducted using the primers M13F and M13R (Table S1) by Macrogen, Inc. (Korea). The sequences from 3′ and 5′ RACE sequencing and the partial cDNA from the clones from the EST database were aligned to identify overlapping regions to generate the full-length cDNA. When the full-length cDNAs of On-CXC1 and On-CXC2 were obtained, the BLASTX and BLASTN programs in the GenBank database (http://www.ncbi.nlm.nih.gov) were used to assess homology against all matched sequences in the database when the E-value was less than 1 × 10^−4^ [42]. The cDNA structure of the open reading frame (ORF) and the 5′ and 3′ untranslated regions (UTRs) of these two cDNAs were analyzed using ORF Finder (Open Reading Frame Finder, http://www.ncbi.nl.nih.gov/gorf/gorf.html). The signal peptide of each CXC protein was analyzed using SignalP software (http://www.cbs.dtu.dk/services/SignalP/).

### 2.6. Homology and Phylogenetic Analyses

The identity and similarity of both nucleotide and amino acid sequences of Nile tilapia CXC cDNA were evaluated against CXC cDNAs of other vertebrates using the MatGAT (Matrix Global Alignment Tool) version 2.02 (http://bitincka.com/ledion/matgat/). The deduced amino acid sequences were aligned and compared with other known CXC chemokine genes of various vertebrate species using the CLUSTAL W program (http://ebi.ac.uk/Tools/clustalw/index.html) [43]. A phylogenetic tree of CXC chemokine genes from vertebrates was constructed using the amino acid sequences of the Nile tilapia CXC chemokine genes and previously reported amino acid sequences of CXC chemokines from other teleosts and vertebrates [34] (See Figure S5). All sequences were aligned using the CLUSTAL W program (http://www.ebi.ac.uk/Tools/clustalw/index.html) [43], and a phylogenetic tree was constructed using MEGA version 6 with 1000 bootstrap replicates [44].

### 2.7. Quantitative Reverse-Transcription Real-Time PCR (qRT-PCR) Analysis for the CXC Chemokine Gene Expression in Various Tissues

#### 2.7.1. Total RNA Isolation and cDNA Synthesis

In this part, total RNA isolated from the brain, gills, gonad, heart, head kidney, intestine, liver, muscle, skin, spleen, stomach, trunk kidney and peripheral blood leukocytes (PBLs) of the healthy fish were used for using TRIzol reagent (Gibco BRL, Carlsbad, CA, USA) according to the manufacturer’s protocol. For PBL isolation, 1.5 mL of whole blood was withdrawn from the caudal vein with a sterile syringe coated with 50 IU heparin solution. The unclotted blood was diluted with 1.5 mL of RPMI medium. This component was further put into a 15-mL conical tube containing 3 mL Histopaque^®^-1077 (Sigma-Aldrich, St Louis, MO, USA). The two-layer mixture was centrifuged at 400× *g* for 30 min at 25 °C in a swing rotor centrifuge, and 3 mL of the opaque interface was aspirated and moved into a new conical centrifuge tube and 3 mL of phosphate-buffered saline (PBS; pH 7.4) was added. The PBL solution was gently mixed and 3 times of centrifugation at 250× *g* for 10 min were made. One millimeter of TRIzol reagent (Gibco BRL, Carlsbad, CA, USA) was added to dissolve the PBL pellet. One hundred milligrams of each tissue in 1 mL TRIzol reagent was carried out as previously described. The contaminating genomic DNA in total RNA samples were treated with DNase I (Fermentas, Waltham, MA, USA). To synthesize first-strand cDNA, a RevertAid First Strand cDNA Synthesis Kit (ThermoFisher, Waltham, MA, USA) was applied in a 200-µL test tube using 1 µg of total RNA from each tissue and an oligo (dT) 18 primer, followed by incubation at 70 °C for 5 min. The first-strand cDNA was carefully synthesized based on the recommendation of the company’s protocol and kept at −20 °C until used.

#### 2.7.2. qRT-PCR

To analyze mRNA levels of the Nile tilapia CXC chemokine transcripts in the 13 tissues from normal fish, qRT-PCR was employed. qRT-PCR reaction was conducted with the same conditions and procedures as described in Section 2.8 with the specific forward primer CXC-1F New and CXC-1R New for *On*-CXC1, and CXC-2F New and CXC-2R New for *On*-CXC2 (Table S1). The relative expression in the brain was used as a calibrator for other tested tissues.

### 2.8. Response Analysis of CXC Chemokine mRNAs of Nile Tilapia to S. agalactiae Using qRT-PCR

#### 2.8.1. Animals

Thirty-three healthy juvenile Nile tilapias (100 g) were used in this assay. Fish were weekly acclimatized in a 250-L fiberglass tank supplemented with fully oxygenated freshwater and were fed a commercial feed at 5% body weight twice a day. At the end of acclimatization, fish were divided into two major groups, where 15 fish for each group were moved into an 80-L glass tank containing clean freshwater with an excellent aeration system. The remaining three fish were used as an initial control group.

#### 2.8.2. Preparation of *S. agalactiae*

The *S. agalactiae* virulent strain (SAAQH001) was obtained from infected Nile tilapia grown in the central region of Thailand. A single colony of *S. agalactiae* was inoculated in 10 mL of tryptic soy broth (TSB, Merck) and incubated in a shaker water bath at 35 °C for 18–24 h. Then, the bacterial cells were collected by centrifugation at 800× *g* for 15 min and washed twice with sterile 0.85% NaCl. The bacterial pellet was then re-suspended in sterile 0.85% NaCl. The *S. agalactiae* concentration was adjusted to 1 × 10^9^ CFU/mL with an optical density at 560 nm of 0.2 and was further serially diluted to reach the target concentration of 1 × 10^3^ CFU/mL.

#### 2.8.3. Experimental Design

In this step, all fish in the first and second groups were intraperitoneally injected with 0.1 mL bacterial suspension containing 1 × 10^3^ and 1 × 10^9^ CFU/mL of *S. agalactiae* prepared in 2.7.2, respectively. The three fish in the control group were injected with 0.1 mL of 0.85% NaCl via the same route as the bacterial injection groups. At 6 h after injection, the head kidney, spleen and trunk kidney were collected from three fish in each group. Additionally, at 12, 24, 48 h and 7 days after injection, the head kidney, spleen and trunk kidney were collected from three fish in each bacterial injected group.

#### 2.8.4. Total RNA Extraction and First-Strand cDNA Synthesis

Total RNA and first-strand cDNA of the head kidney, spleen and trunk kidney were extracted and synthesized from three fish in each group in 2.7.1 at different time points using the same method described previously.

#### 2.8.5. Quantitative Real-Time PCR Assay

First-strand cDNAs from the head kidney, spleen and trunk kidney of the control fish and infected fish from Section 2.8.4 were subjected to quantitative real-time PCR analysis using the Brilliant II SYBR Green qPCR Master Mix (Stratagene, CA, USA) and an Mx3005P real-time PCR system (Stratagene, CA, USA) equipped with analytical software version 4.0 according to the manufacturer’s recommended protocol. Each reaction had a final volume of 12.5 µL and contained 1 µL of first-strand cDNA, 6.25 µL of 2× SYBR Green qPCR Master Mix, 4.75 µL distillated water and 0.5 µL of each specific primer pair, including CXC-1F New and CXC-1R New for *On*-CXC1 amplification or CXC-2F New and CXC-2R New for *On*-CXC2 amplification (Table S1). The CXC chemokine gene expression levels in each sample at different time points were normalized relative to the expression of β-actin using the primers β-actinrealtimeF and β-actinrealtimeR (Table S1). The PCR conditions were 95 °C for 10 min, followed by 40 cycles of 95 °C for 30 s, 55 °C for 1 min and 72 °C for 1 min. DNA melting curve analysis was performed to verify the specificity of the primers. Each sample for both the CXC chemokine and *β-actin* genes were run in triplicate reactions. In order to assess PCR efficiency, a standard plasmid containing the Nile tilapia CXC chemokine and β-actin genes was serially diluted in 10-fold dilution to provide standard curves. The threshold cycles (C_T_) for the CXC chemokine and β-actin genes was calculated, and the standard curve was used to determine their starting copy number. This method is based on equal PCR efficiencies for the target and internal control mRNA. The relative copy number of target mRNA was calculated according to the 2^-∆∆C^_T_ method [45]. Finally, the threshold cycle value difference (C_T_) between the CXC chemokine and β-actin mRNAs of each reaction was used to normalize the level of mRNA and determine their relative expression.

#### 2.8.6. Data and Statistical Analysis

The relative expression ratio of the CXC chemokine mRNAs was calculated in all tested tissues of Nile tilapia in response to *S. agalactiae* at different time points using the expression level of the fish at the initial time as a calibrator. Subsequently, relative expression ratios of CXC chemokine genes at different time courses were analyzed using a one-way analysis of variance (ANOVA). The significance of the means of relative expression ratios was determined using Duncan’s new multiple range test (DMRT) method. Differences were considered significant when the *p*-value was less than 0.05.

### 2.9. Structural Analysis of the CXC Chemokine Genes in the Genomic DNA of Nile Tilapia by Southern Blotting

A DNeasy Blood and Tissue Kit (QIAGEN, Hilden, Germany)) was used to extract genomic DNA from the whole blood of three healthy fish according to the manufacturer’s instructions. Ten micrograms of DNA from each fish as separately used and digested with the *Eco*RI and *Pst*I restriction enzymes following the manufacturer’s protocol (Fermentas, Waltham, MA, USA). The digested DNA was separated on a 1.5% agarose gel in 1× TBE buffer at 50 V. The standard size was based on the reference of a lambda (λ) *Hind* III DNA marker. The digested DNA was transferred to a nitrocellulose membrane by the capillary blotting method with 20× SSC (saline-sodium citrate) using the methods described by Sambrook and Russell [46]. Finally, the obtained membranes were dried at 80 °C for 2 h in a hot-air oven.

The primers CXC-1FSB and CXC-1RSB were used to PCR amplify a specific probe for *On*-CXC1 (Table S1). The PCR probes were labeled with digoxigenin-11-dUTP using a DIG High Prime DNA Labeling and Detection Starter Kit I (Roche Diagnostics, Mannheim, Germany) according to the manufacturer’s protocol. The membrane hybridization and immunological detection procedures was carried out as recommended by the manufacturer. Color detection with NBT/BCIP and analysis was performed to assess the intensity of the bands based on the same methods described in a previous study [47].

### 2.10. Genomic Organization Analysis of the Nile Tilapia CXC Chemokine Genes

The full-length Nile tilapia CXC chemokine sequences (*On*-CXC1 and *On*-CXC2) from the present study were assessed using the Ensembl Nile tilapia genomic databases (www.ensembl.org). BLAST searches were conducted to localize the putative CXC chemokine genes in the Nile tilapia genome. In addition, genomic DNA was extracted from the spleen of a healthy fish as described in Section 2.9. Gene-specific primers targeting exon 1 (CXC1Exon1F) and exon 3 (CXC-1R) (Table S1) of the Nile tilapia CXC chemokine gene (*On*-CXC1) were used to clarify the unclear sequence information found in the Ensembl database. Genomic DNA was used as template for PCR amplification. The PCR analysis was performed with the same conditions described in Section 2.7.2, and the products were purified using a QIAquick^®^ Gel Extraction Kit (Qiagen, Hilden, Germany), ligated into the pGEM-T Easy vector and transformed into JM109 competent cells. Positive colonies were screened by PCR analysis and sequenced as described in Section 2.5.

### 2.11. Overexpression and Functional Analyses of the Nile Tilapia CXC Chemokines

#### 2.11.1. Overexpression of *On*-CXC1 and *On*-CXC2 Chemokine Recombinant Proteins

The cDNAs encoding the mature *On*-CXC1 and *On*-CXC2 chemokine proteins were amplified using the specific primers *On*-CXC1FOverEx and *On*-CXC1ROverEx for *On*-CXC1, *On*-CXC2FOverEx and *On*-CXC2ROverEx for *On*-CXC2) (Table S1) with the following conditions: initial denaturation of 5 min at 95 °C; 25 cycles of 95 °C for 30 s, 55 °C for 30 s and 72 °C for 60 s; and a final elongation step at 72 °C for 5 min. The obtained PCR products were purified using a QIAquick^®^ Gel Extraction Kit (QIAgen^®^), ligated into the pGEM T-Easy vector, and transformed into JM109 competent cells as described in Section 2.5. The plasmid DNAs were extracted, and 1 μL of the prepared plasmid was subjected to double-restriction enzyme digestion (*Nde* I and *Xho* I) to confirm the appearance of the inserted DNAs. The obtained plasmid was sequenced to confirm correct in-frame insertion. The recombinant plasmid was further used for *Nde* I and *Xho* I restriction enzyme digestion, the digested DNA fragments were purified and ligated into the pET28b(+) expression vector and transformed into JM109 competent cells, and the transformed bacteria were grown at 37 °C in LB broth containing 100 mg/mL of kanamycin. The positive clones were extracted and transformed into *E. coli* BL21 (DE3) competent cells for protein expression. After induction, recombinant proteins (r*On*-CXC1 and r*On*-CXC2), containing a His tags at both the N- and C-termini were carefully purified using a previously described protocol [47].

#### 2.11.2. Phagocytic Assays Using Latex Beads as the Substrate

A phagocytosis assay was employed for analyzing the biological activity of the proteins based on the method described by [47], with appropriate modifications. Briefly, PBLs were isolated and pooled from three healthy Nile tilapias as described in Section 2.7.1. The densities of the latex beads (Sigma Aldrich, St Louis, MO, USA) and phagocytic cells were adjusted to 1 × 10^8^ beads/mL and 5 × 10^6^ phagocytes/mL, respectively, with RPMI 1640 medium. Three experimental groups were created for each recombinant protein. For the first and second groups, the latex beads were separately incubated with 10 and 100 µg/mL, respectively, of r*On*-CXC1and r*On*-CXC2 in RPMI medium, while the latex beads in the third (control) group were incubated with RPMI 1640 medium. These groups were run in triplicate. The phagocytic cells adhering to the surface of the cover slip were incubated with the latex beads at different concentrations of protein at room temperature for 1.5 h to allow latex-bead particle uptake. Attached cells and beads were further stained with Diff-Quick staining dye (VR Bioscience Co., Ltd.), and at least 200 cells were counted under 100× light microscopy. The percentage of phagocytosis (PA) and the phagocytic index (PI) were calculated according to the formulas below. Statistical analyses of these two parameters were conducted as described above.

PA = [Number of phagocytic cells with engulfed latex beads/Number of phagocytes] × 100

PI = [Number of engulfed latex beads/Phagocytic cells]

#### 2.11.3. Phagocytic Assays using Viable *S. agalactiae* and *F. columnare* Cells as Substrates

In this experiment, evaluation of the phagocytic activity of PBLs of Nile tilapia mostly followed the protocol described in Section 2.11.2. However, the substrates for phagocytic engulfment were separately replaced with viable cells of *S. agalactiae* and *F. columnare* at the same concentrations. *F. columnare* was prepared in Shieh’s broth based on the protocol described by [47]. Phagocytosis against bacterial cells was visualized and recorded using light microscopy at 1000× magnification at 1, 3 and 6 h, respectively.

#### 2.11.4. Minimal Inhibitory Concentration

The two pathogenic bacteria from Section 2.11.3 were separately used for determination of the minimal inhibitory concentrations (MICs) by liquid growth inhibition methods [48]. Mueller-Hinton broth was used as a test medium in a 96-well microtiter plate. The final concentrations in 200 μL of tested medium (in triplicate) of each r*On*-CXC were serially diluted from 10 to 0.02 μg, and bacterial concentrations of 1 × 10^5^ CFU were used. Wells that only contained broth medium and bacteria were provided as a negative control. After incubation at 30 °C for 24 h, the lowest concentration that caused complete inhibition indicated by clear broth was defined as the MIC. Growth of bacteria in each well was measured at 0, 1, 3, 6, 12, 18 and 24 h with an iMarkTM Microplate Absorbance Reader (Bio-Rad) at 600 nm. During these periods, morphological changes of bacterial cells exposed with 1.25 and 10 mg/mL of each CXC protein were stained using Gram’s staining methods and visualized at 1, 3 and 6 h by light microscopy (Olympus, Woods Hole, MA, USA) under 1000× magnification.

## 3. Results

### 3.1. Characterization of the Nile Tilapia CXC Chemokine cDNAs

In the current study, two full-length cDNAs encoding CXC chemokines in Nile tilapia, which may be alternatively spliced transcripts, were obtained by homology comparisons of EST sequences as reported previously [41]. To obtain the full-length sequences of these cDNAs, 3′ and 5′ RACE was employed. The full-length cDNA encoding the first CXC chemokine of Nile tilapia was named *On*-CXC1 (KR673910) and comprised 1346 bp, including a 408 bp open reading frame (ORF). The predicted amino acid sequence of *On*-CXC1 had 135 amino acid residues and contained a 116 bp 5′ untranslated region (UTR) and an 822 bp 3′UTR. The 3′UTR contained 4 mRNA instability motifs (ATTTA) and 7 different polyadenylation signals, including ACTAAA, TATAAA and five AATAAA sequences (Figure S1). The second cDNA was named *On*-CXC2 (KR673911), and its full-length cDNA was 653 bp, with a 321-bp ORF. The predicted amino acid sequence of *On*-CXC2 had 106 amino acid residues. The complete cDNA of *On*-CXC2 had a 53 bp 5′UTR and a 279 bp 3′UTR. The 3′UTR contained 2 mRNA instability motifs (ATTTA and ATTTTA) and 5 different polyadenylation signals, including one AAAAAG, one AATAAA, one AATAGA and two AAAACA sequences (Figure S2).

In addition, putative signal peptides of both *On*-CXC1 and *On*-CXC2 were predicted at the N-terminus with cleavage between Ile17 and Ser18. Structurally, *On*-CXC1 and *On*-CXC2 had the signature arrangement of four cysteines (C29, C31, C56, C72) that is found in well-known CXC chemokines from other vertebrate species (Figure S3). A single leucine residue separated the first and second cysteines, which is a characteristic CXC chemokine signature found in the CXC chemokine genes of other vertebrates. Interestingly, an ELR (Glutamic acid-Leucine-Arginine) motif preceding the CXC sequence was not found in either *On*-CXC1 or *On*-CXC2. Other conserved amino acids among teleost fish and higher vertebrate CXC chemokines, including isoleucine (position I61) and valine (positions V62, V84), were observed. Structural analysis of the predicted amino acid sequence of the *On-*CXC1 and *On*-CXC2 polypeptides showed that these proteins had a molecular weight and isoelectric point (p*I*) of 15.12 kDa and 9.56 for *On*-CXC1 and 12.11 kDa and 9.76 for *On*-CXC2, respectively.

Moreover, these two sequences were subsequently evaluated for their homology. The results also showed that *On*-CXC1 and *On*-CXC2 had significant amino acid identity and similarity, with scores of 73.7% and 76.3%, respectively (Figure S3).

### 3.2. Comparison of Nucleotide and Amino Acid Sequences and Phylogenetic Analysis

Homology comparison of the deduced amino acid sequences revealed that *On*-CXC1 and *On*-CXC2 shared 13.9–49.1% and 17.3–57.7% sequence identities with CXC chemokines from other fish species and higher vertebrates, respectively. Comparisons of the nucleotide and amino acid sequences of the CXC chemokine genes of Nile tilapia and other organisms revealed that both *On*-CXC1 and *On*-CXC2 showed the highest nucleotide and amino acid sequence identity to *Oplegnathus fasciatus*, with scores of 64.5% and 49.1% for *On*-CXC1 and scores of 70.7% and 57.7% for *On*-CXC2, respectively (Table S2).

The evolutionary relationship of the Nile tilapia CXC chemokine proteins with other known vertebrate CXC chemokines was analyzed using previously reported sequence data [34]. There were 64 CXC chemokine proteins from different species in the phylogenetic tree from 36 higher vertebrates and 28 teleost fish species. The results showed that *On*-CXC1 and *On*-CXC2 were closely related to the Mandarin fish (*Siniperca chuatsi*) CXC chemokine, which was located in a novel CXC subgroup [34]. Moreover, the novel CXC subgroup was shown to be more closely related to the CXCL14 subgroup than any other CXC chemokine subgroup (Figures S4 and S5). In addition, the ELR-containing CXC chemokines (ELR+ subgroup), including CXCL1-7, fish IL-8, and CXCL15, and the mammalian IL-8 subgroup, which had an ELR motif in various species, were strictly separated from the non-ELR-containing chemokines (ELR- subgroup), including CXCL9-14, CXC16, CXCL17 and the novel CXC chemokines.

### 3.3. Genomic Organization of the CXC Chemokine Genes

To determine the genomic organization of the Nile tilapia CXC chemokine genes, a further analysis was conducted by searching in the Ensembl database (www.ensembl.org). The results showed that the *On*-CXC genes matched two different locations in the Nile tilapia genome, scaffolds AERX01074721 (Figure S6A) and GL831305 (Figure S6B). However, the nucleotide sequences were still unclear in some regions, showing many Ns in the sequence files. PCR and DNA cloning followed by sequence analysis were conducted to completely elucidate the sequence information. Interestingly, the complete sequence analysis of scaffold GL831305 revealed that it was approximately 1.6 Mb and contained the full *On*-CXC1 gene sequence, which had 4 exons and 3 introns. Additionally, *On*-CXC2 was observed to share the same first two exons and a partial sequence of exon 3 of *On*-CXC1. However, the gene fragment of a pseudogene comprising part of exon 3, a 250-bp intron, the full exon 4 and the 3′UTR used to generate the complete *On*-CXC2 molecules was observed 24,308 bp upstream of the *On*-CXC1 gene (Figure S6B). Scaffold AERX01074721 (Figure S6A) solely contained 2205 bp of the other pseudogene exon 4 and a partial exon 3 of *On*-CXC1. Structurally, *On*-CXC1 had a 4 exon and 3 intron organization. The exact organization of the *On*-CXC2 gene remains to be characterized.

### 3.4. Structural Analysis of the CXC Chemokine Genes in the Genomic DNA of Nile Tilapia

**Figure 1:**
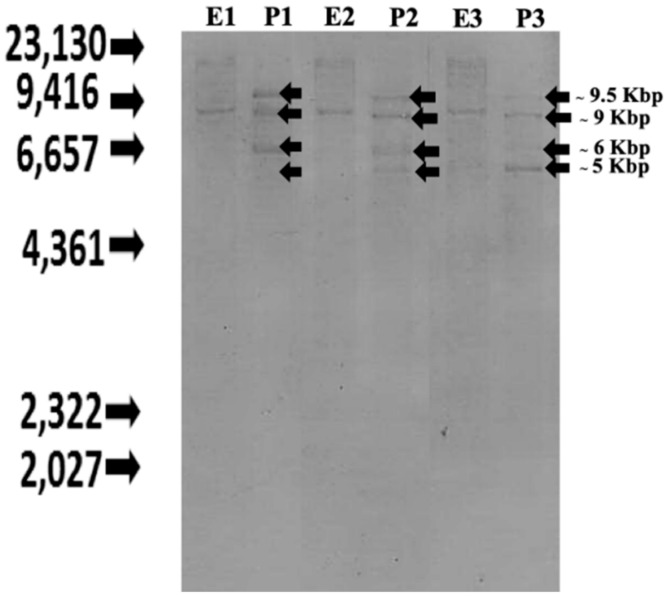
Southern blot analyses of CXC chemokine genes in Nile tilapia genome. Three different genomic DNA samples from three fish *Eco* RI and *Pst* I were digested with *Eco* RI (E1-E3) and *Pst* I (P1-P3), respectively.

To analyze the structure of the Nile tilapia CXC chemokine genes, Southern blotting was performed using the genomic DNA of three individual Nile tilapia. Fish genomes were digested with the *Eco* RI and *Pst* I enzymes. The hybridization of specific probes to the fish genomes showed different patterns, with several hybridized bands. The hybridization patterns of fish genomes digested with *Eco* RI showed similar results at approximately 9 kb. Similarly, the fish genomes digested with *Pst* I also shared four specific bands at approximately 5, 6, 9 and 9.5 kb. These results indicate that there are polymorphisms in the CXC chemokine genes in individual Nile tilapia (Figure 1).

### 3.5. qRT-PCR Analysis of Nile Tilapia CXC Chemokine (On-CXC1 and On-CXC2) mRNAs in Various Tissues of Normal Fish by RT-PCR

**Figure 2:**
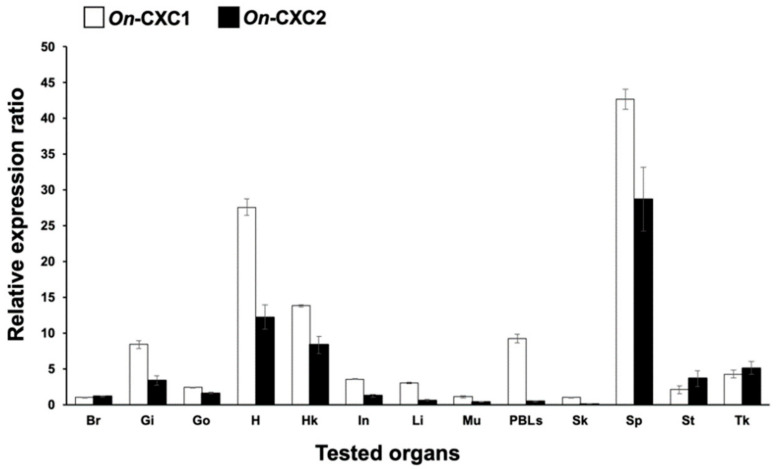
Quantitative RT-PCR analysis of *On*-CXC1 and *On*-CXC2 in various tissues of normal Nile tilapia. Br: brain; Gi: gills; Go: gonad; H: heart; HK: head kidney; In: intestine; Li: liver; Mu: muscle; PBLs: peripheral blood leukocytes; Sk: skin; Sp: spleen; St: stomach; and Tk: trunk kidney. Relative expression values of each gene are presented as the means ± SD, *n* = 3.

Expression analysis of the Nile tilapia CXC chemokine transcripts revealed that both forms were constitutively expressed in all tested tissues of normal fish, including brain, gills, gonad, heart, head kidney, intestine, liver, muscle, PBLs, skin, spleen, stomach and trunk kidney, although slightly lower expression level was observed for the shorter form (On-CXC2) (Figure 2). Interestingly, the highest levels of On-CXC1 were observed in the spleen and heart (27.58 ± 1.15 and 42.66 ± 1.38, respectively), while moderate expression levels were observed in the gills, head kidney and PBLs (8.42 ± 0.54, 13.82 ± 0.18 and 9.26 ± 0.62, respectively). For On-CXC2, the highest expression was found in the spleen (28.75 ± 4.45), and moderate expression was observed in the heart and head kidney (12.25 ± 1.68 and 8.37 ± 1.22, respectively). In addition, On-CXC1 and On-CXC2 mRNAs were expressed at low levels in the brain, gonad, intestine, liver, muscle, skin and stomach (Figure 2).

### 3.6. Expression of On-CXC1 and On-CXC2 in Response to Different Concentrations of S. agalactiae Using Quantitative Real-Time PCR

**Figure 3:**
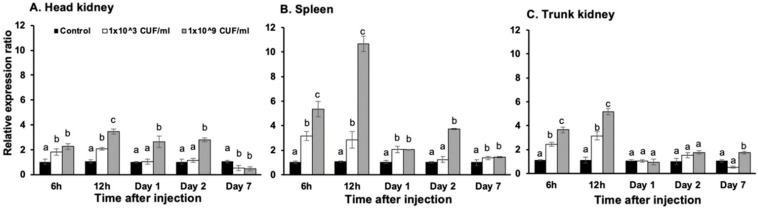
Quantitative real-time PCR analysis of *On*-CXC1 in the head kidney (**A**), spleen (**B**) and trunk kidney (**C**) at different time points in untreated fish and fish injected with 1 × 10^3^ and 1 × 10^9^ CFU/mL of *S. agalactiae.* Different letters (a, b, c) on each bar indicate significant differences at *p* < 0.05.

Real-time quantitative PCR was used to quantify *On*-CXC1 and *On*-CXC2 mRNA expression in Nile tilapia in response to two concentrations of *S. agalactiae* at different time points. In the head kidney, *On*-CXC1 mRNA was significantly increased in fish injected with 1 × 10^9^ CFU/mL of *S. agalactiae* at 6 and 12 h and at days 1 and 2, with relative expression of 2.26 ± 0.24, 3.56 ± 0.22, 2.64 ± 0.46 and 2.78 ± 0.15, respectively, compared to the control (*p* < 0.05). However, at day 7, both concentrations of bacteria significantly decreased mRNA levels of *On*-CXC1 to 0.54 ± 0.22 and 0.48 ± 0.14 compared with the control (*p* < 0.05) (Figure 3A). In the spleen, the higher concentration of *S. agalactiae* significantly elevated the mRNA levels of *On*-CXC1 compared to the other groups at 6 h post-injection; the levels were 5.34 ± 0.61 and reached the highest level of 10.66 ± 0.62 at 12 h (Figure 3B). Similarly, in the trunk kidney at 6 h, 12 h and on day 7, fish exposed to the higher concentration of bacteria exhibited significantly higher mRNA levels compared to the other groups, with levels of 3.66 ± 0.24, 5.16 ± 0.27 and 1.74 ± 0.11, respectively (*p* < 0.05) (Figure 3C).

**Figure 4:**
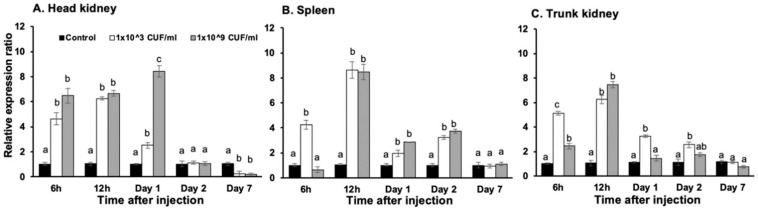
Quantitative real-time PCR analysis of On-CXC2 in the head kidney (**A**), spleen (**B**) and trunk kidney (**C**) at different time points in untreated fish and fish stimulated with 1 × 10^3^ and 1 × 10^9^ CFU/mL of *S. agalactiae*. Different letters (a, b, c) on each bar indicate significant differences at *p* < 0.05.

For *On*-CXC2, at 6 h, 12 h and day 1, the two concentrations of *S. agalactiae* significantly induced mRNA transcript levels in the head kidney compared with other groups, with levels of 4.64 ± 0.48 and 6.48 ± 0.56, 6.26 ± 0.12 and 6.67 ± 0.22, and 2.52 ± 0.22 and 8.42 ± 0.46, respectively (*p* < 0.05). However, at day 7, these bacterial concentrations resulted in significantly lower expression levels than the control with levels of 0.24 ± 0.08 and 0.18 ± 0.14, respectively (*p* < 0.05) (Figure 4A). In the spleen, these two concentrations of bacteria induced significantly higher mRNA expression of *On*-CXC2 than the control at 12 h, 1 and 2 days with 8.64 ± 0.68 and 8.48 ± 0.62, 1.95 ± 0.25 and 2.86 ± 0.04, and 3.25 ± 0.16 and 3.75 ± 0.15, respectively (*p* < 0.05) (Figure 4B). In the trunk kidney, *On*-CXC2 transcripts were enhanced by the lower concentration of *S. agalactiae* at 6 and 12 h and days 1 and 2 and were higher than those of the control by 5.12 ± 0.16, 6.25 ± 0.35, 3.25 ± 0.12, and 2.55 ± 0.22, respectively (*p* < 0.05). The higher concentration of *S. agalactiae* effectively increased the *On*-CXC2 mRNA level compared to the control only at 6 and 12 h with 2.45 ± 0.16 and 7.45 ± 0.27, respectively (*p* < 0.05) (Figure 4C).

### 3.7. Functional Analyses of rOn-CXC1 and rOn-CXC2 Proteins

**Figure 5:**
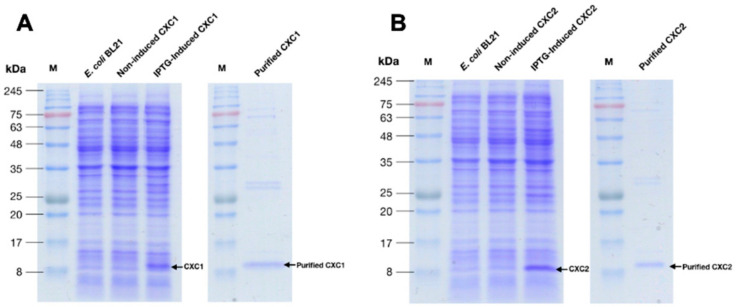
Overexpression of the recombinant proteins and purification of *On*-CXC1 (r*On*-CXC1) (**A**) and *On*-CXC2 (r*On*-CXC2) (**B**). M is the protein marker.

The recombinant proteins of both r*On*-CXC1 and r*On*-CXC2 were successfully produced as soluble forms. The purified r*On*-CXC1 (Figure 5A) and r*On*-CXC2 (Figure 5B) were approximately 12–14 kDa. These two recombinant proteins were used to further analyze their biological functions.

**Figure 6:**
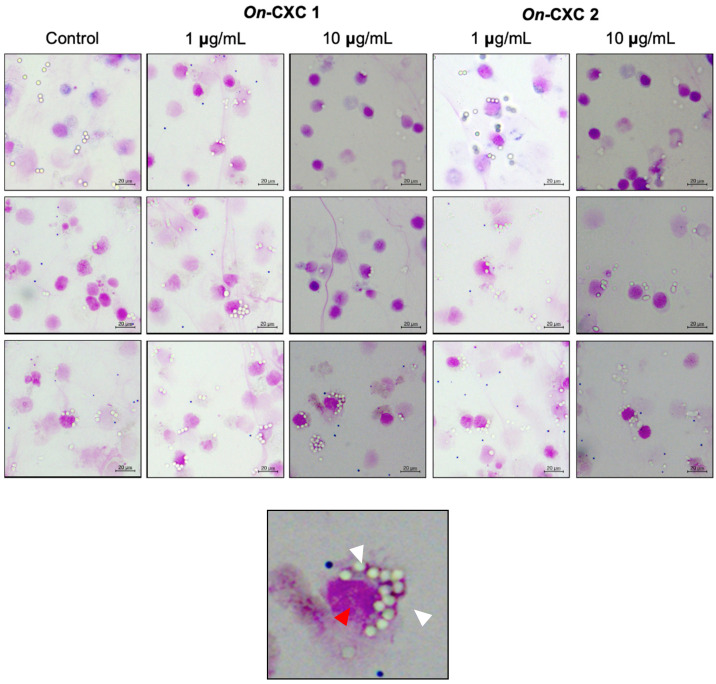
Effects of r*On*-CXC1 and r*On*-CXC2 at 1 and 10 mg/mL on the phagocytic activity of PBLs using latex beads as the targeting substrates. White arrows in a zoom-in image (below) indicate engulfing latex beads in a phagocytic cell (red arrow).

**Figure 7:**
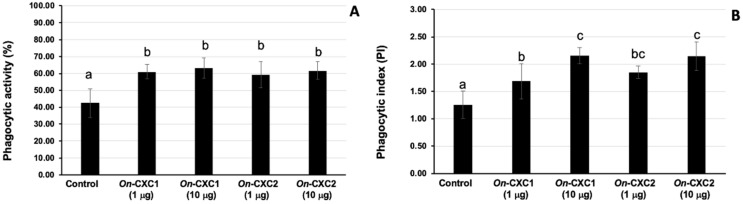
Phagocytic activity (**A**) and phagocytic index (PI) (**B**) of PBLs enhanced with 1 and 10 μg of r*On*-CXC1 and r*On*-CXC2. Different letters (a, b, c) on each bar indicate a significant difference at *p* < 0.05.

The r*On*-CXC1 and r*On*-CXC2 proteins at concentrations of 1 and 10 μg clearly enhanced both PA and PI, as indicated by the bead-engulfing cells (Figure 6). The PA of chemokine-treated PBLs exhibited values ranging from 59.21 ± 7.65–63.15 ± 5.94%, which were significantly higher than those of the control (42.50 ± 8.54%) (*p* < 0.05) (Figure 7A). Similarly, the concentrations of both r*On*-CXC1 and rOn-CXC2 proteins strongly elevated the PI, with values of 1.69 ± 0.32–2.16 ± 0.15, which significantly differed from the control, with a PI value of 1.26 ± 0.25 (*p* < 0.05) (Figure 7B).

**Figure 8:**
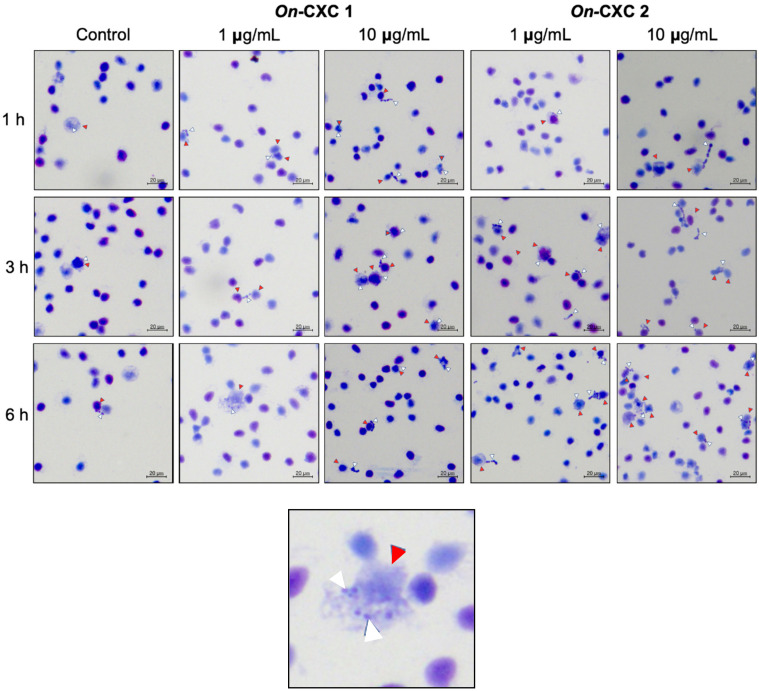
Effects of r*On*-CXC1 and r*On*-CXC2 at 1 and 10 μg/mL on the phagocytic activity of PBLs against *S. agalactiae* cells during a 1–6 h exposure. Red and white arrows indicate a PBL and bacterial cells, respectively.

**Figure 9:**
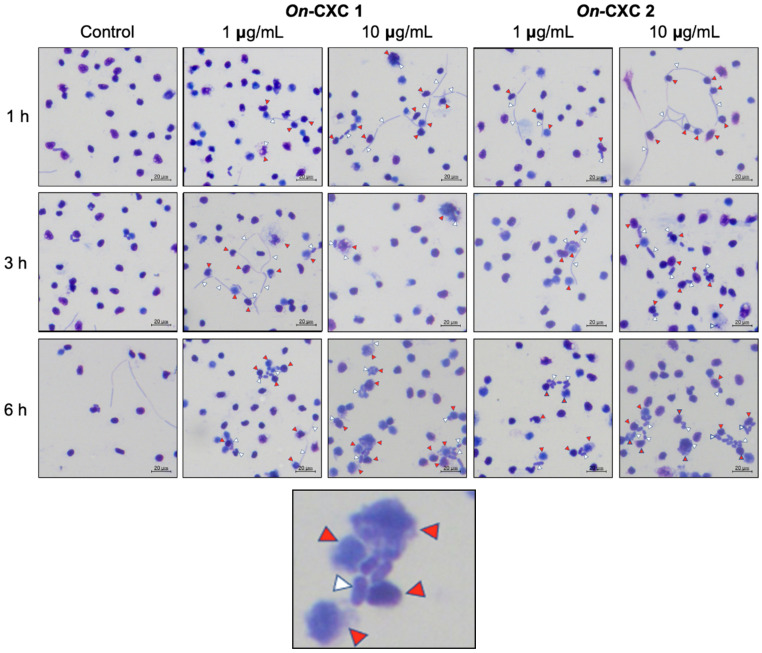
Effects of r*On*-CXC1 and r*On*-CXC2 at 1 and 10 μg/mL on the phagocytic activity of PBLs against *F. columnare* cells during a 1–6 h exposure. Red and white arrows indicate a PBL and bacterial cells, respectively.

Phagocytosis against viable pathogenic bacteria was also detected in the r*On*-CXC1 and r*On*-CXC2 protein-treated PBLs. Several engulfed streptococci and long, rod-shaped cells were observed. Unexpectedly, morphologically changed cells were normally found to be engulfed, especially at 3 and 6 h. Swollen streptococci and mono- and diplococcus of *S. agalactiae* (Figure 8) and shortened rod shapes or cell debris of *F. columnare* were commonly observed in PBLs in the 10 μg-CXC treated groups (Figure 9).

**Figure 10:**
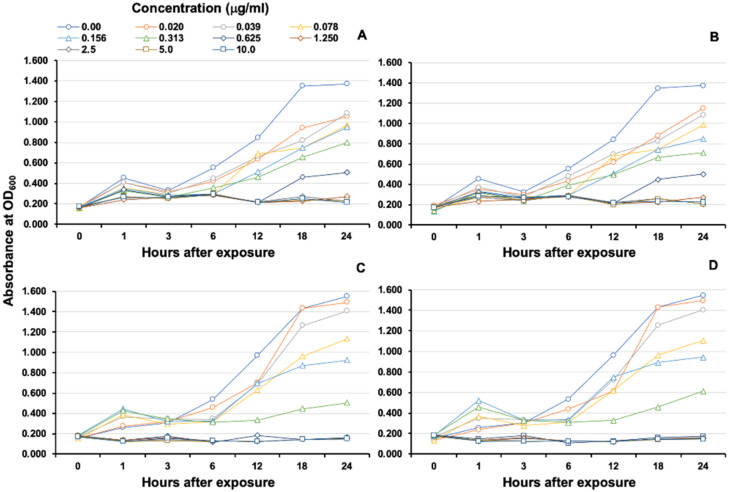
MIC and growth curve analyses of *S. agalactiae* and *F. columnare* after exposure to r*On*-CXC1 and r*On*-CXC2 at 0–10 μg/mL. *S. agalactiae* exposed to r*On*-CXC1 and r*On*-CXC2, respectively (**A**,**B**). *F. columnare* exposed to r*On*-CXC1 and r*On*-CXC2, respectively (**C**,**D**). The absorbance was measured at OD_600_ for different concentrations and time courses.

**Figure 11:**
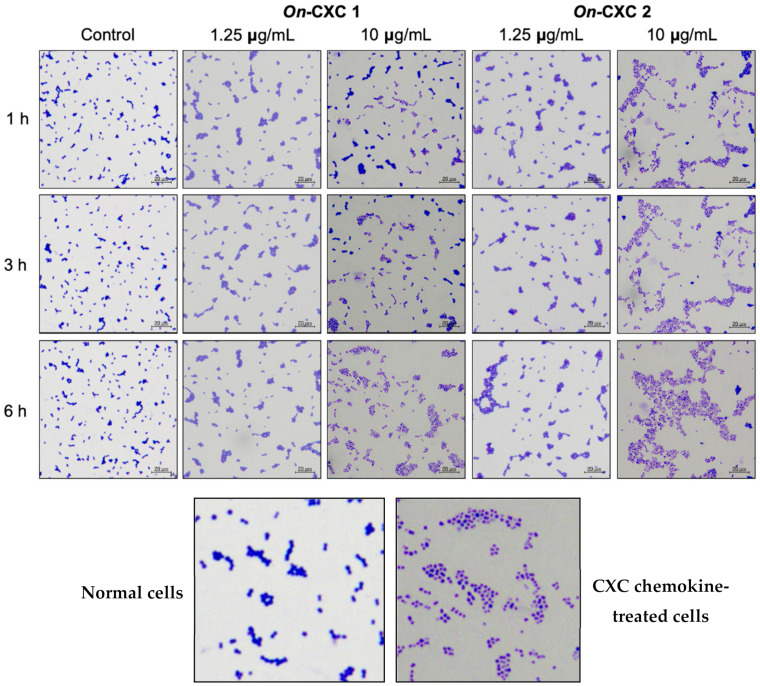
Effects of r*On*-CXC1 and r*On*-CXC2 at 1.25 and 10 μg/mL on *S. agalactiae* cells during a 1–6-h exposure. Below pictures showing zoom-in images of normal and CXC chemokine treated cells.

**Figure 12:**
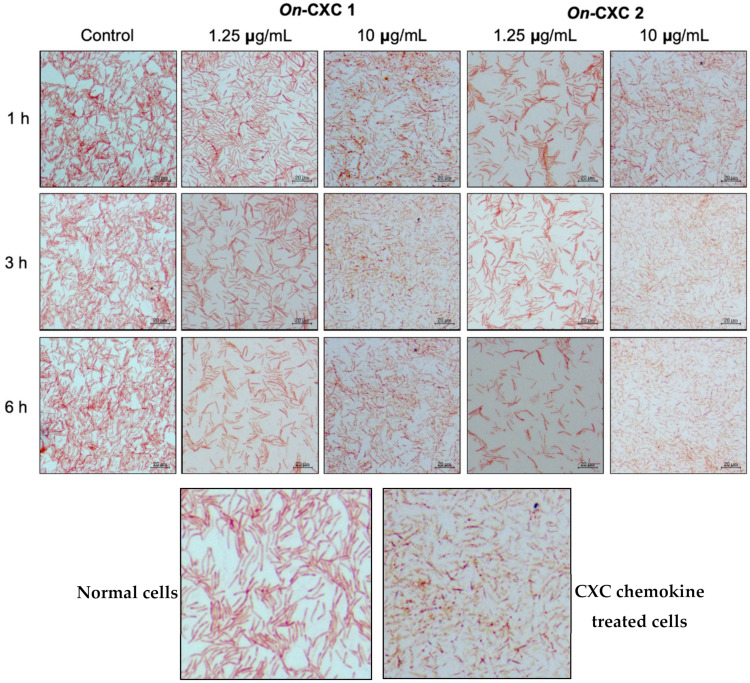
Effects of r*On*-CXC1 and r*On*-CXC2 at 1.25 and 10 μg/mL on *F. columnare* cells during a 1–6 h exposure. Below pictures showing zoom-in images of normal and CXC chemokine treated cells.

The MICs of r*On*-CXC1 and r*On*-CXC2 proteins against *S. agalactiae* and *F. columnare* were very similar. The r*On*-CXC1 and r*On*-CXC2 exhibited MICs of 0.313 μg/mL when tested with *S. agalactiae* (Figure 10A,B) and 0.156 μg/mL when tested with *F. columnare* (Figure 10C,D). Interestingly, *S. agalactiae* exposed to these proteins from 1–6 h exhibited cell swelling and abnormal cell wall staining, especially at 10 μg/mL at 6 h (Figure 11). Similarly, r*On*-CXC1 and r*On*-CXC2 at 10 μg/mL strongly affected *F. columnare* by completely lysing most bacterial cells observed from 3–6 h (Figure 12).

## 4. Discussion

Previously, we also successfully characterized and functionally analyzed CC chemokines from Nile tilapia at both the genomic and proteomic levels [47]. Regarding the results of this study, it was clearly shown that the *On*-CXC chemokines have many differences from the *On*-CC chemokines with respect to their gene organization, transcription and functional attributes.

In the present study, two full-length cDNAs encoding Nile tilapia CXC chemokines were successfully cloned and named *On*-CXC1 and *On*-CXC2. Structurally, several characteristics in the putative *On*-CXC sequences identified them as members of the CXC chemokine family. The deduced *On*-CXC1 and *On*-CXC2 sequences possessed the typical arrangement of four cysteine residues found in other CXC chemokines. These four cysteine residues were crucial for the formation of the tertiary structure and consequently the effector functions of the molecules. Furthermore, the two N-terminal cysteines of CXC chemokines were separated by one non-conserved amino acid residue (Leu30), creating a typical CXC motif. The presence of putative signal peptides in both *On*-CXC1 and *On*-CXC2 suggests that Nile tilapia CXC chemokines are secreted molecules, as reported in other vertebrate chemokines.

Information from the Nile tilapia genome and sequence analysis of the obtained cDNAs clearly showed that *On*-CXC1 had 4 exons and 3 introns, which is consistent with previous studies in humans, catfish, rainbow trout and soft-shelled turtles [49] (Figure S6), while *On*-CXC2 was generated by a specific process from a pseudogene fragment containing Ψ exons 3-4 and some part (exons 1 and 2) of the functional *On*-CXC1 gene. This pattern has not been previously reported in any vertebrates. Gene duplication of the pseudo Ψ*CXC* gene fragments may be specific to Nile tilapia or other teleosts that occurred during their evolution and is important for generating diverse gene products and modulating gene expression and function in vertebrate species, including teleost fish. In recent years, alternative splicing analyses of CXC chemokine genes have been reported in several fish genomes, such as the zebrafish, medaka, fugu, channel catfish and stickleback [31,50] indicating the significant role of alternative splicing in teleost immune systems. Therefore, an unknown splicing mechanism used to create the *On*-CXC2 in this study may be a unique process that additionally helps Nile tilapia to produce other forms of CXC molecules in association with invading pathogen defense mechanisms. Further study is needed to elucidate the associated mechanism.

Potential instability motifs (ATTTA) were also identified in the 3′UTR of *On*-CXC1 and *On*-CXC2, similar to previously reported CXC chemokines in other teleosts. This motif is typically found in inflammatory genes, including cytokines, chemokines and oncogenes, and indicates a short half-life. This motif also contributes to the rapid and accurate degradation of mRNA for the regulation of gene expression [51]. It suggests that these two transcripts are unstable, as has been observed with other chemokines. Interestingly, the Nile tilapia CXC transcripts contained both a typical polyadenylation signal sequence (AATAAA) and atypical polyadenylation sequences (ACTAAA, TATAAA, AAAAAG, AAAACA and AATAGA), which are also found in rainbow trout [27], catfish [52], Japanese flounder [31], and large yellow croaker [33]. Essentially, polyadenylation is the last key step in the mRNA maturation process, which is important for the translation efficiency, stability, and localization of mRNA, while alternative polyadenylation has a specific functional role to the generation of distinct mRNA isoforms from the same gene [53]. This suggested that these transcripts may add a poly (A) tail at one of several possible sites to create other transcripts of differently functional molecules.

Structurally, the Nile tilapia CXC chemokine molecules found in this study lacked an ELR motif immediately preceding the CXC motif. Therefore, they were classified into the non-ELR subgroup and identified as novel CXC chemokines. Unlike IL-8 molecules in mammals and avian species, the Nile tilapia CXC chemokines in the present study and other fish CXC chemokines, with the exception of haddock and Atlantic cod CXC molecules, lack an ELR motif immediately preceding the CXC motif. Several studies have identified the ELR motif in various species and shown that ELR-containing CXC chemokines attract neutrophils, whereas non-ELR-containing chemokines mainly attract lymphocytes and monocytes [16,17]. However, the immunological functions of the non-ELR Nile tilapia CXC chemokines remain unclear compared to other teleost fish. Therefore, further investigations are required to determine the biological activities and immunological functions of these molecules.

The amino acid sequence and phylogenetic analysis of the 64 CXC chemokine members of fish and higher vertebrates indicated separately clustering of 10 different subgroups. Both *On*-CXC1 and *On*-CXC2 were placed into a novel CXC subgroup. Therefore, they were grouped with the Mandarin fish, rock bream and Atlantic halibut CXC chemokines and clustered with the closely related Mandarin fish CXC chemokines. In addition, the novel CXC chemokine subgroup was strictly separated into a distinct clade compared to related genes in other teleosts and higher vertebrates. Therefore, they may be derived from different evolutionary events than those of other teleosts and higher vertebrates. This information may provide further insight into the evolutionary relationship between chemokines of Nile tilapia and other species.

In this study, the gene expression profiles of the Nile tilapia CXC chemokines were analyzed by qRT-PCR. The qRT-PCR results demonstrated that both transcripts were constitutively expressed at different levels in all tested tissues of normal fish. A constitutive expression pattern of CXC chemokine genes has also been found in several fish, such as catfish [52], Japanese flounder [31], common carp [29] and large yellow croaker [33]. These results indicate that the CXC chemokines, which are produced by resident tissue macrophages in all body compartments of fish, may have a possible homeostatic function in immunosurveillance by constitutive recruitment of leukocyte populations under normal physiological conditions [54]. In addition, the highest expression level of *On*-CXC1 and *On*-CXC2 was observed in the spleen, which is one of the most important hematopoietic tissues. These results suggest that the spleen is an important site of lymphocytes and macrophages, which are sources of CXC chemokines under normal conditions.

Interestingly, slightly different levels of the two different *On-*CXC transcripts were observed in all the tested tissues of normal fish. This result may have been due to the ability of the *On-*CXC1 and *On-*CXC2 that continuously modulate gene expression and function under normal conditions [50]. Therefore, further studies are necessary to assess variations in CXC chemokine expression (*On*-CXC1 and *On*-CXC2) to understand the regulation of this mechanism.

Upon induction with *S. agalactiae*, real-time PCR was used to quantify Nile tilapia CXC mRNA expression in the immune-related organs of the fish, including head kidney, spleen and trunk kidney. The kidney (anterior and posterior) and spleen are the largest lymphoid organs in teleost fish and are the source of macrophages involved in the phagocytosis of bacteria in innate immunity. Therefore, the basal expression of cytokines and chemokines is most prominent in these tissues. The result from this study showed that *On*-CXC1 and *On*-CXC2 mRNA were rapidly up-regulated to different levels in all tested tissues during the first 6–12 h after injection with 1 × 10^3^ and 1 × 10^9^ CFU/mL of *S. agalactiae*. During this time, the CXC chemokines might be vigorously produced by monocytes and macrophages in the infected site to eliminate foreign antigens; there, they function as the immunomodulators of leukocytes. Similar to other fish, non-ELR-containing CXC chemokines appear to be regulated by bacterial stimuli. The elevated expression of non-ELR-containing CXC chemokines following induction by different pathogens or immune-enhancing components has been reported in several teleost species, such as catfish challenged with *Edwardsiella ictaruli* [55], common carp after stimulation with LPS and concanavalin A (ConA) [56] and rock bream after stimulation with LPS and polyinosinic-polycytidylic acid (poly I:C) [34].

In this study, the Nile tilapia CXC chemokine genes were abundantly expressed in the spleen and trunk kidney, which are classified as secondary lymphoid tissues in teleosts. Generally, streptococcosis targets the brain and eyes as well as the spleen and kidney, which contain a large number of immune cells, including lymphocytes and professional macrophages [57]. Therefore, the strong induction of Nile tilapia CXC chemokines after exposure to *S. agalactiae* in this study indicated their important role as chemoattractants that recruit inflammatory cells to the infected site.

Based on the dose-dependent induction, the concentration of 1 × 10^9^ CFU/mL of *S. agalactiae* was shown to be a potent inducer of CXC chemokines in Nile tilapia, while the concentration of 1 × 10^3^ CFU/mL showed a lesser degree of induction. These results indicate that 1 × 10^9^ CFU/mL of *S. agalactiae* strongly stimulates CXC chemokine expression compared to lower concentrations of pathogenic bacteria. This could be due to the speed of disease progression between different concentrations of an invading pathogen.

Moreover, different expression patterns of *On*-CXC1 and *On*-CXC2 were observed in this study. This was especially apparent in the trunk kidney, where 1 × 10^3^ CFU/mL of *S. agalactiae* induced higher *On*-CXC2 mRNA levels from 6 h to 7 days compared to 1 × 10^9^ CFU/mL of *S. agalactiae*. This may have been due to different mature mRNA isoforms generated by the alternative splicing mechanism, which resulted in the appearance of different functions or expressions in different cell types or tissues. Many recent studies have reported that alternative splicing is the major mechanism for producing diverse gene products and modulating gene expression [50]. In catfish, the alternative splicing of IL-8-like genes [16] resulted in normal and alternatively spliced forms being expressed at different levels. This suggests that alternative splicing is an important mechanism for modulating gene expression and function of fish IL-8 genes [50,58], similar to the CXC genes in Nile tilapia. However, the expressions of both Nile tilapia CXC chemokine genes in this study were decreased to baseline levels at day 2. This suggests a suppressive role of CXC chemokines on their transcription by feedback inhibition via membrane proteins (chemokine receptors). Thus, they have a short half-life in response to this pathogen and are produced as needed, which are characteristics of cytokine and chemokine genes found in other vertebrates [59]. Additionally, the shorter transcript *On*-CXC2 was more sensitive to both lower and higher concentrations of *S. agalactiae* at 12 h than *On*-CXC1 in all tested tissues, and it also sensitively responded to low doses of bacteria in the trunk kidney from days 1–7, suggesting that the shorter CXC protein may have specific responses to the concentration of stimulated bacteria, organs and reaction times.

Southern blot analysis and further analyses using the Ensembl project (www.ensembl.org) and PCR amplification were conducted to elucidate the genomic organization of these genes. These results clearly confirmed the presence of the chemokine gene components and pseudogenes involved in the novel mechanisms of CXC production in Nile tilapia. Generally, the alternative splicing is an important mechanism for generating diverse gene products and modulating gene expression and functions in eukaryotic species, including some CXC chemokines of teleosts. However, the mechanism used to create the *On*-CXC2 was not observed to produce the CXC proteins in other vertebrates. Obviously, the genomic organization of Nile tilapia CXC chemokines, which contain four exons and three introns, was consistent with well-defined fish CXC chemokine gene structure, and the splice sites for the first two introns have also been conserved. However, the process used to generate the *On*-CXC2 by using pseudogene fragments and the functional *On*-CXC1 gene and the variation in a splice site in the third intron of the *On*-CXC1 gene may reflect a divergence event that led to differences in the structure and size of these genes. Further information from other teleosts may aid in supporting and elucidating this unusual process to diversify of CXC genes and its transcriptional and translational processes.

Several recombinant CXC chemokine proteins have been studied for their biological activity in a variety of fish species, such as carp [60], rock bream [34], trout [61] and channel catfish [62]. In this study, r*On*-CXC and r*On*-CXC2 were also assessed for their phagocytic activities, which are the important roles of most chemokine proteins. It has been well documented that phagocytosis is one of the most important processes of the innate immune response against microbial infection and/or tissue injury [63]. The results in this study demonstrated that r*On*-CXC1 and r*On*-CXC2 significantly enhanced the PA and PI of phagocytes in PBLs. Cells treated with 1 and 10 µg/mL r*On*-CXC1 and r*On*-CXC2 exhibited increased phagocytic activity, suggesting that all concentrations of r*On*-CXC1 and r*On*-CXC2 were sufficient to elevate the phagocytic activity of phagocytes in vitro. Moreover, the highest level of PI was shown in the experimental group treated with 10 µg/mL of both r*On*-CXC1 and r*On*-CXC2, indicating that this concentration can improve the efficiency of CXC chemokines to stimulate phagocytes by enhancing the engulfment capacity.

Additionally, the effects of r*On*-CXC1 and r*On*-CXC2 on the phagocytic activity using *S. agalactiae* and *F. columnare* target cells were also investigated. The results clearly demonstrated that phagocytic activity was enhanced by r*On*-CXC1 and r*On*-CXC2 in a dose- and time-dependent manner. The result of this experiment revealed the positive correlation with phagocytosis using latex beads. In addition, the morphology changes of both *S. agalactiae* and *F. columnare* indicated the antimicrobial activities of these CXC chemokines.

In the current study, the capacities of Nile tilapia CXC chemokines to mediate antimicrobial activity against pathogenic bacteria were analyzed. The results showed that both r*On*-CXC1 and r*On*-CXC2 have broad-spectrum antimicrobial activities acting against both Gram-positive (*S. agalactiae*) and -negative (*F. columnare*) bacteria in a dose-dependent manner. Interestingly, the chemokines showed slightly stronger antimicrobial activity against *F. columnare* compared with *S. agalactiae*. Our findings are consistent with several reports where LPS, lipid A, or lipoteichoic acid could be a target for the bactericidal activity of AMPs [64,65]. This may reflect the fact that the antimicrobial mechanisms, which consist of membrane depolarization and permeabilization of these Nile tilapia CXC chemokines with Gram-positive and Gram-negative bacteria, may be different. However, the differences in mechanism of action on Gram-positive and -negative bacteria of these Nile tilapia CXC chemokines are still not clear. Based on current information, the defense mechanism of Gram-positive bacteria may protect against AMPs by increasing the positive charge of the bacterial cell surface through the addition of D-alanine to wall teichoic and lipoteichoic acids [66,67,68], while the extracellular membranes of Gram-negative bacteria contain a layer of lipopolysaccharide (LPS), which imparts a strongly negative charge. In addition, cationic AMPs can bind to LPS, causing cell membrane damage, lysis and death [69].

Due to the electrostatic interactions between cationic chemokines and the anionic bacterial cell membrane, the morphological changes of *S. agalactiae* and *F. columnare* cells were obviously different from the untreated group, suggesting that Nile tilapia CXC chemokines may attach to the negative charges on bacterial cell walls to damage bacterial cell membranes and release cell fluid. The results in this experiment revealed the correlation with antimicrobial activity, which showed a decline in bacterial cell growth in the CXC-treated group compared with the untreated group. Following exposure to r*On*CXC1 and r*On*CXC2, obvious morphological changes occur in *F. columnare* cells. At 10 µg/mL, elongated cells and shortened cells were observed for short periods, and lysed cells were observed after exposure for long (3–6 h) periods. In the case of *S. agalactiae*, the bacterial cells changed in shape (swelling or irregular shape) and clumped together, changes that were associated with cell membrane destruction and loss of intracellular constituents. These findings suggested that different types of bacteria may affect different reactions of the CXC chemokines.

## 5. Conclusions

In summary, analyses of the molecular immunology and immunological effects of bacterial infection on Nile tilapia CXC genes contribute to our understanding of fish immune responses. These data provide basic molecular information useful for further investigations of the function of CXC chemokine genes in the Nile tilapia immune system and the evolutionary relationships of important immune-related genes among vertebrates. Information obtained in the present study clearly indicates a dual function of Nile tilapia CXC chemokines as the mediators of antimicrobial activity. Additionally, the chemokines could initially promote leukocyte migration to sites of infection and simultaneously kill pathogenic bacteria. Hence, these properties of CXC chemokines will facilitate bacterial clearance and an enhanced immune responses in fish, which will be useful for developing effective strategies, such as highly potent vaccine adjuvants for disease prevention from the harmful pathogenic bacteria that are now causing mass mortality in the Nile tilapia industry.

## Supplementary Materials

The following are available online at https://www.mdpi.com/2076-2607/8/7/1058/s1, Figure S1: Nucleotide and deduced amino acid sequences of the Nile tilapia CXC chemokine (*On*-CXC1) Cdna; Figure S2: Nucleotide and deduced amino acid sequences of the Nile tilapia CXC chemokine (On-CXC2) cDNA; Figure S3: Sequence alignment between On-CXC1 and On-CXC2; Figure S4: Multiple sequence alignment of On-CXC1and On-CXC2 with other known CXC chemokines from various species; Figure S5: Phylogenetic tree showing the relationships between the Nile tilapia CXC chemokine (*On*-CXC1, *On*-CXC2) amino acid sequences and other known CXC chemokines from various species; Figure S6: Genomic structure and organization comparison of the Nile tilapia CXC chemokine genes with other known CXC chemokines of other vertebrates; Table S1: Oligonucleotide primers used in this study; Table S2. Homological analyses of nucleotide and amino acid sequences of On-CXC1 and On-CXC2 against CXC chemokines of other vertebrates.
