# Supplementary material for: Molecular Identification and Dual Functions of Two Different CXC Chemokines in Nile Tilapia (Oreochromis niloticus) against Streptococcus agalactiae and Flavobacterium columnare
Source: Microorganisms. 2020 Jul 16;8(7):1058. doi: 10.3390/microorganisms8071058 (PMC7409096; doi:10.3390/microorganisms8071058)
Supplement: Supplementary file 1 [file microorganisms-08-01058-s001.pdf]

Primer name	Nucleotide sequence (5’→3’)	Purpose
CXC-1F New	TGAACCCTGAGCTGAAGGCCGTGA	Real-time PCR
CXC-1R New	TGAAGGTCTGATGAGTTTGTCGTC	Real-time PCR
CXC-1R	CCTTCAGCTCAGGGTTCAAGC	Genomic PCR
CXC-2F New	GCTTGAACCCCGAGCTGAAAAACG	Real-time PCR
CXC-2R New	GTTCAGAGGTCGTATGAGGTGCTT	Real-time PCR
CXC-2F	CAAGCAGGACAACAGTGTCTGTGT	3’RACE
CXC-2AR	GTTGCATGATTTGGATGCTGGGTAG	5’RACE
CXC-1FSB	AACATATGTCTCCCAGGCCCAACTCAAAC	Southern blot
CXC-1RSB	CTCGAGTTATTTTGCACTGATGTGCAA	Southern blot
CXC1Exon1F	CAAAGTGTTTCTGCTCCTGG	Genomic PCR
On-CXC_1FOverEx	CATATGCAACTCAAACAAGCAGGACAACAGT	Overexpression
On-CXC_1ROverEx	CTCGAGTTTTGCACTGATGTGCAATTTCAA	Overexpression
On-CXC_2FOverEx	CATATGCAACTCAAACAAGCAGGACAACAGT	Overexpression
On-CXC_2ROverEx	CTCGAGCATGGCAGCTGTGGAGGGTTCCAC	Overexpression
β-actinrealtimeF	ACAGGATGCAGAAGGAGATCACAG	Real-time PCR
β-actinrealtimeR	GTACTCCTGCTTGCTGATCCACAT	Real-time PCR
M13F	AAAACGACGGCCAG	Nucleotide sequencing
M13R	AACAGCTATGACCATG	Nucleotide sequencing
UPM-long (0.4 µM)	CTAATACGACTCACTATAGGGCAAGCAGTGGTATCAACGCAGAGT	RACE PCR
UPM-short (2 µM)	CTAATAC GACTCACTATA GGGC	RACE PCR

Table S1. Nakharuthai and Srisapoome (2020)

On-CXC1	Nucleotide (%)	Amino acid (%)		*On*-CXC2	Nucleotide (%)	Amino acid (%)	
Versus	identity	identity	similarity	Versus	identity	identity	Similarity
**Teleost fish**							
1. Rock bream, *Oplegnathus fasciatus* (AB703273)	64.5	49.1	68.1	*O. fasciatus*	70.7	57.7	75.4
2. Mandarin fish, *Siniperca chuatsi* (AAY79282)	63.2	48.1	68.9	*S. chuatsi*	70.5	54.0	78.8
3. Atlantic halibut, *Hippoglossus hippoglossus* (ACY54778)	52.0	39.3	51.1	*H. hippoglossus*	64.5	46.3	63.9
4. Common carp IL-8, *Cyprinus carpio* (ABE47600)	44.9	19.1	34.1	*C. carpio*	49.4	21.9	42.6
5. Rainbow trout IL-8, *Oncorhynchus mykiss* (CAC33585)	44.0	21.3	36.3	*O. mykiss*	47.3	23.7	44.4
6. Japanese flounder IL-8, *Paralichthys olivaceus* (AAL05442)	48.4	25.4	45.9	*P. olivaceus*	49.6	29.6	55.0
7. Atlantic cod IL-8, *Gadus morhua* (CAD59734)	42.2	21.5	37.0	*G. morhua*	47.6	21.4	46.3
8. Haddock IL-8, *Melanogrammus aeglefinus* (CAD97422)	43.4	20.1	38.5	*M. aeglefinus*	48.4	23.9	48.1
9. Fugu rubripes IL-8, *Takifugu rubripes* (NP001027759)	45.0	20.6	37.0	*T. rubripes*	50.3	24.6	47.2
10. Black porgy IL-8, *Acanthopagrus schlegelii* (AAY18807)	42.1	18.8	38.5	*A. schlegelii*	44.1	24.3	48.1
11. European seabass IL-8, *Dicentrarchus labrax* (CAM32186)	44.0	19.6	37.0	*D. labrax*	48.5	23.3	45.4
12. Bighead, *Hypophthalmichthys nobilis* (JN546631.1)	44.8	22.0	38.5	*H. nobilis*	46.2	25.4	48.1
13. Channel catfish CXCL10, *Ictalurus punctatus* (AAQ01586)	43.5	22.5	43.7	*I. punctatus*	46.8	27.0	55.6
14. Rainbow trout CXCL10, *Oncorhynchus mykiss* (NP001117788)	44.3	22.0	43.7	*O. mykiss*	46.7	26.3	54.6
15. Zebrafish CXCL11, *Danio rerio* (XP001339307)	42.2	20.6	40.0	*D. rerio*	46.0	24.8	50.0
16. Atlantic salmon CXCL10, *Salmo salar* (NP001134500)	38.3	23.7	37.0	*S. salar*	42.9	27.7	44.4
17. Nile tilapia CXCL14, *Oreochromis niloticus* (XP003443444)	43.3	21.4	37.8	*O. niloticus*	48.5	28.4	43.5
18. Atlantic salmon CXCL14, *Salmo salar* (NP001134297)	44.2	20.0	36.3	*S. salar*	44.6	26.7	41.7
19. Rainbow trout CXCL14, *Oncorhynchus mykiss* (CCD11011)	45.6	20.0	36.3	*O. mykiss*	46.5	26.7	41.7
20. Atlantic salmon CXCL13, *Salmo salar* (NP001134571)	46.7	19.0	41.5	*S. salar*	47.1	21.1	44.1
21. Japanese flounder CXC, *Paralichthys olivaceus* (BAF79875)	46.8	22.9	43.7	*P. olivaceus*	47.7	28.1	51.4
22. Fugu rubripes CXCL13, *Takifugu rubripes* (NP001233223)	41.4	21.3	39.3	*T. rubripes*	49.4	23.9	48.1
23. Zebrafish CXCL12, *Danio rerio* (NP840092)	44.0	17.1	38.5	*D. rerio*	46.4	20.4	49.1
24. Large yellow croaker CXCL12, *Larimichthys crocea* (ACF35274)	43.8	13.9	35.6	*L. crocea*	46.4	17.3	44.4

**Higher vertebrates**							
25. African clawed frog CXCL14, *Xenopus laevis* (NP001086198)	45.8	20.0	41.5	*X. laevis*	47.8	21.2	44.4
26. African clawed frog CXCL12, *Xenopus laevis* (CAC82196)	43.5	18.7	47.7	*X. laevis*	47.7	21.4	51.9
27. Cattle IL-8, *Bos taurus* (NP_776350)	46.6	17.9	38.5	*B. taurus*	47.0	22.1	49.1
28. Sheep IL-8, *Ovis aries* (NP_001009401)	47.6	17.1	38.5	*O. aries*	46.7	21.2	49.1
29. Pig IL-8, *Sus scrofa* (NP999032)	47.9	17.1	37.8	*S. scrofa*	47.5	21.2	48.1
30. Dog IL-8, *Canis lupus familiaris* (NP001003200)	47.2	17.9	38.5	*C. lupus*	48.7	21.2	49.1
31. Domestic cat IL-8, *Felis catus* (NP001009281)	46.5	17.9	37.0	*F. catus*	45.8	21.2	46.3
32. Horse IL-8, *Equus caballus* (NP001077420)	46.5	19.3	34.8	*E. caballus*	46.6	23.0	44.4
33. Human IL-8, *Homo sapiens* (NP000575)	44.3	16.4	36.3	*H. sapiens*	46.3	20.4	46.3
34. Rhesus monkey IL-8, *Macaca mulatta* (NP001028137)	45.7	17.9	37.0	*M. mulatta*	43.9	21.2	47.2
35. Chicken IL-8, *Gallus gallus* (NP990829)	43.9	23.0	39.3	*G. gallus*	48.1	27.7	49.1
36. House mouse CXCL1, *Mus musculus* (NP032202)	43.4	18.6	36.3	*M. musculus*	45.7	21.2	45.4
37. Human CXCL3, *Homo sapiens* (NP002081)	42.4	18.4	39.3	*H. sapiens*	46.2	20.8	50.0
38. Human CXCL11, *Homo sapiens* (NP005400)	40.6	23.3	45.2	*H. sapiens*	44.7	26.3	51.9
39. Horse CXCL10, *Equus caballus* (NP001108412)	47.7	23.0	43.7	*E. caballus*	48.9	27.7	54.6
40. Pig CXCL10, *Sus scrofa* (ABD18444)	46.1	21.2	42.2	*S. scrofa*	47.1	25.5	52.8
41. Rhesus monkey CXCL13, *Macaca mulatta* (NP001028052)	44.3	17.3	40.7	*M. mulatta*	49.9	21.2	47.7
42. HumanCXCL13, *Homo sapiens* (NP006410)	44.6	17.3	40.7	*H. sapiens*	46.7	20.4	47.7

Table S2. Nakharuthai and Srisapoome (2020)

nt1	CGAATCCTCACAGCACTCCTGTCAACAATCAGTCTTTAATTGTTGCTAGTCTTTCTCTGG	nt60
nt61	CTGCATTATTATCTTTGCTGCACGTTTTCAGCACCGGTGTCATCTTGAAAACAACA**ATG**T	nt120
aa1	**M** S	aa2
nt121	CCACCCTCATCAAAGTGTTTCTGCTCCTGGCTGTCATGGTCTGCATCTCCCAGGCCCAAC	nt180
aa3	T L I K V F L L L A V M V C I S Q A Q L	aa22
nt181	TCAAACAAGCAGGACAACAGTGTCTGTGTCACCGTGTCAGGAATCGTCTCGGCATGAAGT	nt240
aa23	K Q A G Q Q C L C H R V R N R L G M K S	aa42
nt241	CTGAAATAAAGGACGTACAGATCTACCCAGCAACCAAATTCTGCAACAAAGTGGAGATTG	nt300
aa43	E I K D V Q I Y P A T K F C N K V E I V	aa62
nt301	TCGTTACCTTGAACAGTGGTCTCCGCTATTGCTTGAACCCTGAGCTGAAGGCCGTGAAAA	nt360
aa63	V T L N S G L R Y C L N P E L K A V K R	aa82
nt361	GACTCGTGACTAACATCATGGATAAAAAGCAGAAGACCACTTCCTCCCCAGTGGAACCCT	nt420
aa83	L V T N I M D K K Q K T T S S P V E P S	aa102
nt421	CCACAGCTGCCAATATAATTTTGACGACAAACTCATCAGACCTTCATATCATTTGTAGTA	nt480
aa103	T A A N I I L T T N S S D L H I I C S I	aa122
nt481	TTTTTAAAGAATATTGCTTGAAATTGCACATCAGTGCAAAA**TAA**TCAGCATTACTAAAAT	nt540
aa123	F K E Y C L K L H I S A K *	aa135
nt541	ATGTCTCAGGGAAATGGAAAATATTCAAGAATTTTCCACTTGAATTAGTCCTTTAACATA	nt600
nt601	ATGAAACACAGCAGCAACATTTAGCACATGCAAACAGCACAGCACAGCCAGTCACACACA	nt660
nt661	GTCCTGCTATACATAACAAGAGGTAGAGGCTAAAGAAGACAAAATCAAACAAACAAACAA	nt720
nt721	GACAGTTGATTTTAAGCAGGTTTCAGTTGCTGAAAAAAATGATATTCTAGAAATATGAAG	nt780
nt781	GTATGACACCAAAGGATGGTTTCCCTCTTGGAGCATGAATGTTCCCTCTAATTCTGAATT	nt840
nt841	TCATTAATTTGACCCCTGAGATGATTTTCATTTATTCTTTAGTCCTGCTCTAGTTATACT	nt900
nt901	TATTTATACTTTTTACTTATATATTTAATTAGTCCCTTCAGGATTTTACTCTCTGTATTC	nt960
nt961	CACTTTGTCCTCCTCCCATGTTAAGTTTAATTCATGTGTAAGCATTTCCCTGCTGCAGTT	nt1020
nt1021	GTTGTTATTCATGTCCCACTTTAGTTTTGTAATAATTTGTCTTCTGTGTTTCATGTTTAA	nt1080
nt1081	TTTTACTTCACGTCCTAGCTCATTGCCATTGTCTTCAACTGTGTGATTACCTTGTGTCTG	nt1140
nt1141	TTTATCCTCCTGTTTTCAATCTCCTGACATGTGTGTATAAATAGTCCTGTCTATGATCCA	nt1200
nt1201	TTTGGCAAAATAAATCCATTTGGTATCCTTGTTGGTCTTCAGACAACAATTCTGACGGCT	nt1260
nt1261	TAAGAACCAAATGGGACAGACAAAAAAAATAAAAAATAAATAAATAAATAAATAAATAGT	nt1320
nt1321	CCTGTCAAAAAAAAAAAAAAAAAAAA	nt1346

Figure S1. Nakharuthai and Srisapoome (2020)

nt1	ACATGGGATCTTTGCTGCACGTTTTCAGCACCGGTGTCATCTTGAAACCAACA**ATG**TCCA	nt60
aa1	**M** S T	aa3
nt61	CCCTCATCAAAGTGTTTCTGCTCCTGGCTGTCATGGTCTGCATCTCCCAGGCCCAACTCA	nt120
aa4	L I K V F L L L A V M V C I S Q A Q L K	aa23
nt121	AACAAGCAGGACAACAGTGTCTGTGTCACCGTGTCAGGAATCGTCTCGGCATGAAGTCTG	nt180
aa24	Q A G Q Q C L C H R V R N R L G M K S E	aa43
nt181	AAATAAAGGACGTACAGATCTACCCAGCAACCAAATCATGCAACAAAGTGGAGATTCTCG	nt240
aa44	I K D V Q I Y P A T K S C N K V E I L V	aa63
nt241	TTACCTTCAACAGTGGTCTCCGCTATTGCTTGAACCCCGAGCTGAAAAACGTGAAAAGAC	nt300
aa64	T F N S G L R Y C L N P E L K N V K R L	aa83
nt301	TCGTGACTAACATCATGGATAAAAAGCAGAAGACCACTTCCTCCCCAGTGGAACCCTCCA	nt360
aa84	V T N I M D K K Q K T T S S P V E P S T	aa103
nt361	CAGCTGCCATG**TGA**TTGTCTTTTAAGCTGCCTGAAAGAAAAAGCACCTCATACGACCTCT	nt420
aa104	A A M *	aa106
nt421	GAACCCTCAACAAAGTCACTTTGACAAAAACAAAAAAAACAAACATGGACATGAAATGAT	nt480
nt481	TGTAGTTTTTTTTATTTAATTTTTTTATACATGCTTTTATTTTATCTCCACTCTTTTGTA	nt540
nt541	TCCTGTTTTTATTCTACTGTGTTTTTGTAAATAGACGAATGCCTCTTTCTATCTTTTGAT	nt600
nt601	ATTTTTTTAATAAAATTTCTAACTCTAAAAAAAAAAAAAAAAAAAAAAAAAAA	nt653

Figure S2. Nakharuthai and Srisapoome (2020)

```
On-CXC1      MSTLIKVFLLLAVMVCISQAQLKQAGQQCLCHRVRNRLGMKSEIKDVQIYPATKFCNKVEIVVTLNSGLRYCLN
On-CXC2      MSTLIKVFLLLAVMVCISQAQLKQAGQQCLCHRVRNRLGMKSEIKDVQIYPATKSCNKVEILVTFNSGLRYCLN
             ****************************************************** ******:**:*********

On-CXC1      PELKAVKRLVTNIMDKKQKTTSSPVEPSTAANIILTTNSSDLHIICSIFKEYCLKLHISAK
On-CXC2      PELKNVKRLVTNIMDKKQKTTSSPVEPSTAAM-----------------------------
             **** **************************
```

On-CXC1	Nucleotide	Amino acid	
VS	Identity (%)	Identity (%)	Similarity (%)
On-CXC2	76.0	73.7	76.3

Figure S3. Nakharuthai and Srisapoome (2020)

```
CattleIL-8                  ----------MTS-----------KLAVALLAAFLLSAALCEAAVLSRMS-----------TELRCQCIKTHSTPFHPKFIKELRVIESGPHCENSEIIVKLT-----NGNEVCLNPKEKWVQKVVQVFVKRAEKQDP------------ 101
SheepIL-8                   ----------MTS-----------KLAVALLAAFLLSAALCEAAVLSRMS-----------TELRCQCIKTHSTPFHPKFIKELRVIESGPHCENSEIIVKLT-----NGKEVCLDPKEKWVQKVVQAFLKRAEKQDP------------ 101
DogIL-8                     ----------MTS-----------KLAVALLAAFVLSAALCEAAVLSRVS-----------SELRCQCIKTHSTPFHPKYIKELRVIDSGPHCENSEIIVKLF-----NGNEVCLDPKEKWVQKVVQIFLKKAEKQDP------------ 101
PigIL-8                     ----------MTS-----------KLAVAFLAVFLLSAALCEAAVLARVS-----------AELRCQCINTHSTPFHPKFIKELRVIESGPHCENSEIIVKLV-----NGKEVCLDPKEKWVQKVVQIFLKRTEKQQQQQ---------- 103
Domestic_catIL-8            ----------MTS-----------KLVVALLAAFMLSAALCEAAVLSRIS-----------SELRCQCIKTHSTPFNPKLIKELTVIDSGPHCENSEIIVKLV-----NGKEVCLDPKQKWVQKVVEIFLKKAEKQNA------------ 101
HorseIL-8                   ----------MTS-----------KLAVALLAVFLLSAALCEAAVVSRIT-----------AELRCQCIKTHSKPFNPKLIKEMRAIESGPHCENSEIIVKLV-----NGAEVCLNPHTKWVQIIVQAFLKRAEGQNP------------ 101
HumanIL-8                   ----------MTS-----------KLAVALLAAFLISAALCEGAVLPRSA-----------KELRCQCIKTYSKPFHPKFIKELRVIESGPHCANTEIIVKLS-----DGRELCLDPKENWVQRVVEKFLKRAENS-------------- 99
Rhesus_monkeyIL-8           ----------MTS-----------KLAVALLAAFLLSAALCEGAVLPRSA-----------KELRCECIKTYSKPFHPKFIKELRVIESGPHCANTEIIVKLS-----DGRELCLDPKEPWVQRVVEKFVKRAENQNP------------ 101
ChickenIL-8                 ----------MNG-----------KLG-AVLALLLVSAALSQGRTLVKMG-----------NELRCQCISTHSKFIHPKSIQDVKLTPSGPHCKNVEIIATLK-----DGREVCLDPTAPWVQLIVKALMAKAQLNSDAPL--------- 103
Common_carpIL-8             ----------MHC-----------KIFLVSVIVFLGFLTTGEGMSLRGLG-----------VDPRCRCIETESRRIG-KHIESVELFPPSSHCKDTEIIATLKI----SGKEICLDPTAPWVKKVIEKIIANKAP--------------- 98
Bighead                     ----------MNC-----------KIFSVFVTVAVAFLTISEGMSLRGLG-----------VDLRCRCIKTESRRIG-KHIESVELYPPSPHCKDTEIIATLKE----GKQEICLDPTAPWVKKVIEKILANKAP--------------- 98
Zebra_fishIL-8              ----------MTS-----------KIISVCVIVFLAFLTIIEGMSLRGLA-----------VDPRCRCIETESRRIG-KHIKSVELFPPSPHCKDLEIIATLMT----TGQEICLDPSAPWVKKIIDRIIVK------------------ 95
Rainbow_troutIL-8           ----------MSI-----------RMSASLVVVLLALLTITEGMSLRGMG-----------ADLRCRCIETESRRIG-KLIKKVEMFPPSSHCRDTEIIATLSK----SGQEICLDVSAPWVKRVIEKMLANNK---------------- 97
Black_porgyIL-8             ----------MSS-----------RVFVATIVGLLAFLAISE----ASLG-----------VELHCRCIQTESKPIG-RHIEKVELIPANSHCEETEIIATLKR----TGQEVCLDPEAPWVKKVIQKILSNARR--------------- 94
European_seabassIL-8        ---------MMSS-----------KVFATSIVVLLAFLAISEGMSLRSLG-----------VELHCRCIQTESKPIG-RHIGKVELIPANSHCEETEIIATLKK----TGQEVCLDPEAPWVKKVIQKILSNRRR--------------- 99
Fugu                        ----------MCS-----------RVFLTSLVVLLAFLAISNGMSLRSLG-----------VEQHCRCIQTESRPIG-RHIGKVELIPPNSHCEETEIIATLKM----SGQEVCLDPKAPWVKKVINKIMSSRQR--------------- 98
Japanese_flounderIL-8       ----------MSS-----------RVIVVAVMVLLASLAISEAVSLRSLG-----------VSLHCRCIETESRPIG-RYIKSVEIISPNSHCDKTEIIATLKD----TGVEICLDPEAPWVKRVINKLISKRRLSRWREMGSEAV---- 109
Atlantic_codIL-8            --------MKMTSG----------KIPIGSLLVLLVLLTITEGRSLRGLG-----------MELRCRCIQTESRQIG-RHIGMVEIIPANSHCEETEIIATLKR----TGQEVCLDADAPWVKNVIERMISSRRH--------------- 101
HaddockIL-8                 --------MKMTSG----------KIPISSLLVLLVLLSITEGKSLRGLG-----------MELRCRCIQTESRPIG-RHIGKMEIIPANSHCEESEIIATLKR----TGQEVCLDGEAPWVKRLIAKMMSSRRR--------------- 101
HumanCXCL3                  --MAHATLSAAPSN---------PRLLRVALLLLLLVAASRRAAGASVVT------------ELRCQCLQTLQ-GIHLKNIQSVNVRSPGPHCAQTEVIATLK-----NGKKACLNPASPMVQKIIEKILNKGSTN-------------- 107
PigCXCL2                    --MASAAIAASPCA---------PRFLRAALLLLLLVAAGRRTAGAPVGG------------ELRCQCLQTVQ-GIHLKNIQDLKVTSPGPHCDQTEVIATLK-----NGQEVCLNPAAPMVKKIIEKMLNKSSAN-------------- 107
House_mouseCXCL1            ---------MIPAT---------RSLLCAALLLL---ATSRLATGAPIAN------------ELRCQCLQTMA-GIHLKNIQSLKVLPSGPHCTQTEVIATLK-----NGREACLDPEAPLVQKIVQKML-KGVPK-------------- 96
HumanCXCL7                  ---MSLRLDTTPSCNSARPLHALQVLLLLSLLLTALASSTKGQTKRNLAKGKEESLDSDLYAELRCMCIKTTS-GIHPKNIQSLEVIGKGTHCNQVEVIATLK-----DGRKICLDPDAPRIKKIVQKKLAGDESAD------------- 128
House_mouseCXCL4            MSVAAVFRGLRPS----------PELLLLGLLFLPAVVAVTSAGPEESDG------------DLSCVCVKTISSGIHLKHITSLEVIKAGRHCAVPQLIATLK-----NGRKICLDRQAPLYKKVIKKILES------------------ 105
Channel_catfishCXCL10       ----------------------MKSAAVFVV-FACLLIVHVQGQARTSVR--------------RCLCQGPAANGVRLQRIDKIEIHPASATCENKEIIVTLKN----GAGKKCLNPESEFTKKYITAALEKRSAV-------------- 95
Rainbow_troutCXCL10         ------------------MTNTMTSTVLISF-LACLLLANVEGQVGHSKA--------------RCQCQNGLVNRVKPLHIEKLEVYTSSHSCRNMEIIVTLKN----GEEKKCLNPEAPFAKKTIEKIMKKQRSVQ------------- 100
ZebrafishCXCL11             -----------------------MKTVTALL-LVSLAVVAIEGQHMKSQ---------------RCVCLGAGLNMVKPVLIEKIEILPSSPSCGHMEVIATLKN----GAGKRCLNPKSKFTKKIIDKIEKNNRNAR------------- 94
HumanCXCL11                 ---------------------MSVKGMAIAL-AVILCATVVQGFPMFKRG--------------RCLCIGPGVKAVKVADIEKASIMYPSNNCDKIEVIITLKE----NKGQRCLNPKSKQARLIIKKVERKNF---------------- 94
Atlantic_salmonCXCL10       ----------------------MRTATLILL-CVTVFGAGFAQFPGGRSE--------------KCLCRGKLMQSVRIKRIQKLEVYPNSVFCAKTEIIATMKN----GK-KKCLNPEGKLGKRFLMRKR-------------------- 88
HorseCXCL10                 ---------------------MNQSAVLILC-LIFLTLSGTQGIPLSRTA--------------RCTCINISDRPIPPRSLEKLEMIPASQSCQRVEIIATMKK----NGEKRCLNPESKTVKNLLKAISKQRS---------------- 94
PigCXCL10                   ---------------------MNQSAVLIFC-LILLTLSGTQGIPLSRTV--------------RCTCIKISDRPVNPRSLEKLEMIPASQSCPHVEIIATMKK----NGEKRCLNPESKAIKNLLKAISKERS---------------- 94
HumanCXCL9                  ---------------------MKKSGVLFLLGIILLVLIGVQGTPVVRKG--------------RCSCISTNQGTIHLQSLKDLKQFAPSPSCEKIEIIATLK-----NGVQTCLNPDSADVKELIKKWEKQVSQKKKQKNGKKHQKKKV 110
Atlantic_salmonCXCL14       ----------------------MHRCSTAALLLLIVALYS----LQAEAY--------------KCRCTRKGPKIRY-KDVQKLEIKPKHPFCQEKMIFVTMENVSRFKGQEYCLHPKLQSTKNLVKWFRI----WKDKHRVYEA----- 100
Rainbow_troutCXCL14         ----------------------MHRCTTAALLLLIIALYS----LQAEAY--------------KCRCTRKGPKIRY-KDVQKLEIKPKHPFCQEKMIFVTMENVSRFKGQEYCLHPKLQSTKNLVKWFRI----WKDKHRVYEA----- 100
Nile_tilapiaCXCL14          ----------------------MRGCTT-VLLLLMVALCC----LSAEAY--------------KCRCTRKGPKIRY-KDVQKLEIKPKHPFCQEKMIFVTMENVSRFKGQEYCLHPKLQSTKNLVKWFRI----WKDKHRVYEA----- 99
African_clawedfrogCXCL14    -----------------------MRPITAALILLLVAVST----LHVEGS--------------KCKCSRKGPKIRF-TDVQKLEIKPKYPYCEERMIIVTMQNVSRFRGQQYCLHPKLHSTKKFLKWYTI----WKDKNRVYED----- 99
Rhesus_monkeyCXCL13         ---------------------MKFISASLLLMLLVSSLSPVQGVLEVYYTH------------LRCRCVQESSVFIPRRFIDRIQISPRGNGCPRKEIIVWKK-----NKSVVCVDPQAEWIQRIMEMLRKKSSSTPPVPVFKRKIP--- 109
HumanCXCL13                 ---------------------MKFISTSLLLMLLVSSLSPVQGVLEVYYTS------------LRCRCVQESSVFIPRRFIDRIQILPRGNGCPRKEIIVWKK-----NKSIVCVDPQAEWIQRMMEVLRKRSSSTLPVPVFKRKIP--- 109
Japanese_flounderCXC        ----------------------MMKLQKPLLLLAALTLCCCIDTLHAFRQ-------------RGCHCIRTTPDKVPVRFIKKLEVIPISGQCRRTEIIITKR-----NGYKLCVAPEEKWIKDLLGYLQSENETTDKTSISTTHSPAPD 110
Fugu_rubripesCXCL13         ------------------------MMVKPPTLLVVMTLCCCLITADAFF---------------GCHCLRTIRKPIPLNVIEKIEMLPISGHCRRPEIILTRK-----NGSKICIDPNQKWFKDLLNKMQKEGERP-----SSTTAPV-N 100
Atlantic_salmonCXCL13       ------------------------MLFKPHYLLVVLTLCC-FAALHAFPMGGFA-------PRRTCRCIRTSSAFISPMRFHKLEILPAGSHCSRIEIIVTKK-----DKTIVCVNPEARWINKVIALLQRNKASAG-VPISTTTDGINT 112
HumanCXCL12                 --------------------MNAKVVVVLVLVLT--ALCLSDGKPVSLSY--------------RCPCRFFES-HVARANVKHLKILN-TPNCA-LQIVARLKN----NNRQVCIDPKLKWIQEYLEKALNKRFKM-------------- 93
African_clawedfrogCXCL12    --------------------MDIRTLALLSILLG--TLCLTEGKPVSLVY--------------RCPCRYFES-NVPKSNIKHLKILS-TSNCS-LQIVARLKH----NGKQICLDPKTKWIQEYLEKALNKKAKKT------------- 94
ZebrafishCXCL12             --------------------MDLKVIVVVALMAVAIHAPISNAKPISLVE--------------RCWCRSTVN-TVPQRSIRELKFLH-TPNCP-FQVIAKLKN----N-KEVCINPETKWLQQYLKNAINKMKKAQQQQV--------- 99
Large_yellow_croakerCXCL12  --------------------MDVKLLAVVVAFMV-IYAPPSQAKPISLVE--------------RCYCRSTVN-NIPRGYIRELRFIH-TPNCP-FQVIAKLKT----S-KEVCVNPEIRWLQQYLKNAINKMKRSKQGN---------- 97
Mandarin_fish               -----------------------MSSIMKVFLLLAVMVCISKAQLHQSGQ--------------RCLCNRIRSKLAFKSEVKDIQIYPVNIFCNKVEIVVTLK-----RGFRYCLDPKLDSMKKLLANIIK-QK-TSTTARPTELTSTPG 106
Rock_bream                  -----------------------MSGILKVFLLLAVMVCISKAQLHESGQ--------------QCLCRSVRKGIDSKTKVKDIQIYPATAFCDKVEIVVTNN-----SGLRYCLDPKMQTVKKLVANVMK-QKRSSTTVRPTEFTSTSG 107
Atlantic_halibut            -----------------------MSGIMKVLLLLAASVCISTALLNESGQ--------------NCLCQRTYNSTDG-SDLKDIQIYPATIFCDKVEIVVTTG-----AGHRYCLNHRAKAVKAIVIRILR---SKRNAITPPQSS---- 100
On-CXC2                     -----------------------MSTLIKVFLLLAVMVCISQAQLKQAGQ--------------QCLCHRVRNRLGMKSEIKDVQIYPATKSCNKVEILVTFN-----SGLRYCLNPELKNVKRLVTNIMETDKKQKTTSSPVEPSTAAM 108
On-CXC1                     -----------------------MSTLIKVFLLLAVMVCISQAQLKQAGQ--------------QCLCHRVRNRLGMKSEIKDVQIYPATKFCNKVEIVVTLN-----SGLRYCLNPELKAVKRLVTNIMD--KKQKTTSSPVEPSTAAN 106
                                                                                             * *                        *    ::               *:         :
```

Figure S4. Nakharuthai and Srisapoome (2020)

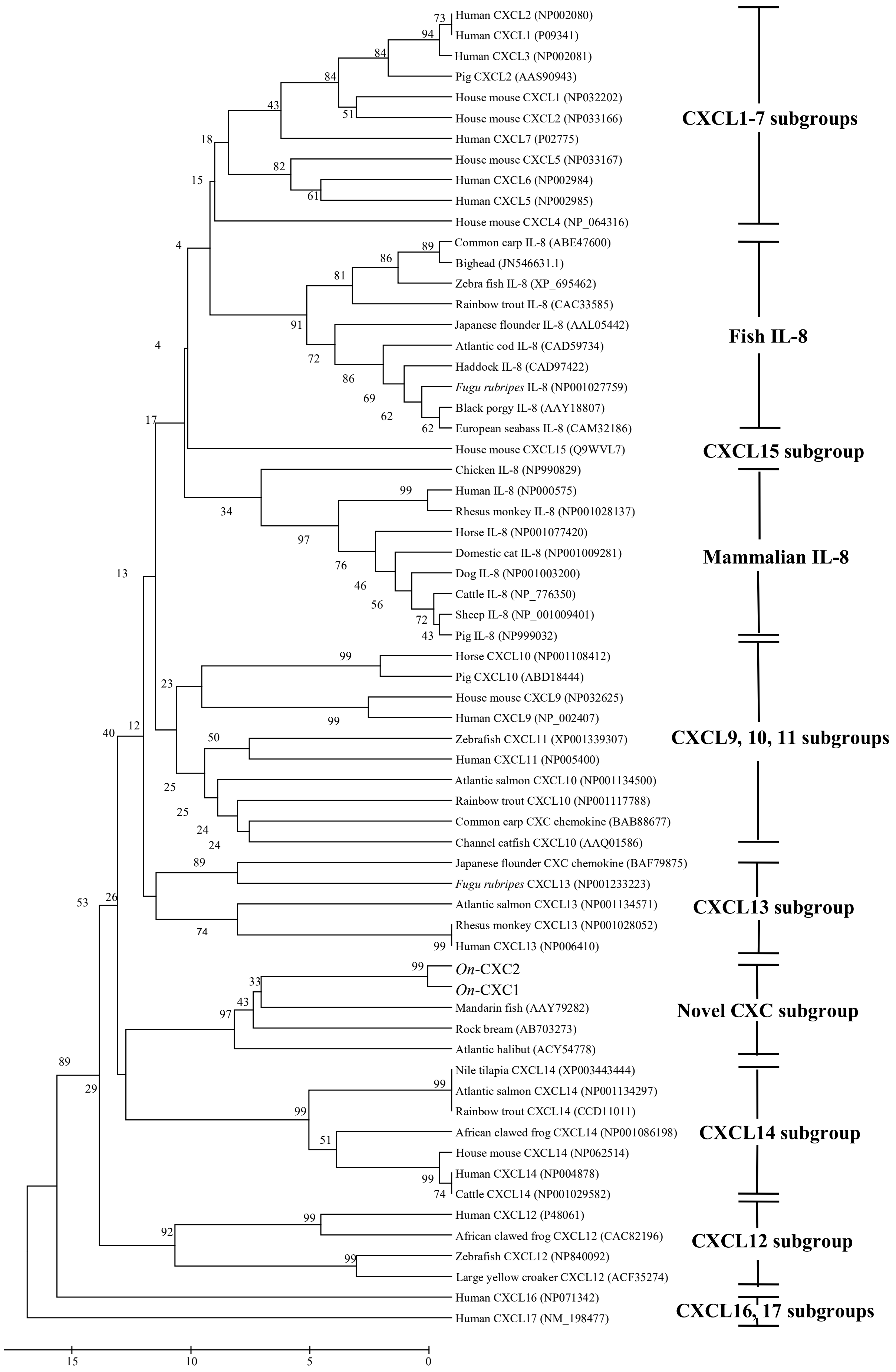

Figure S5. Nakharuthai and Srisapoome (2020)

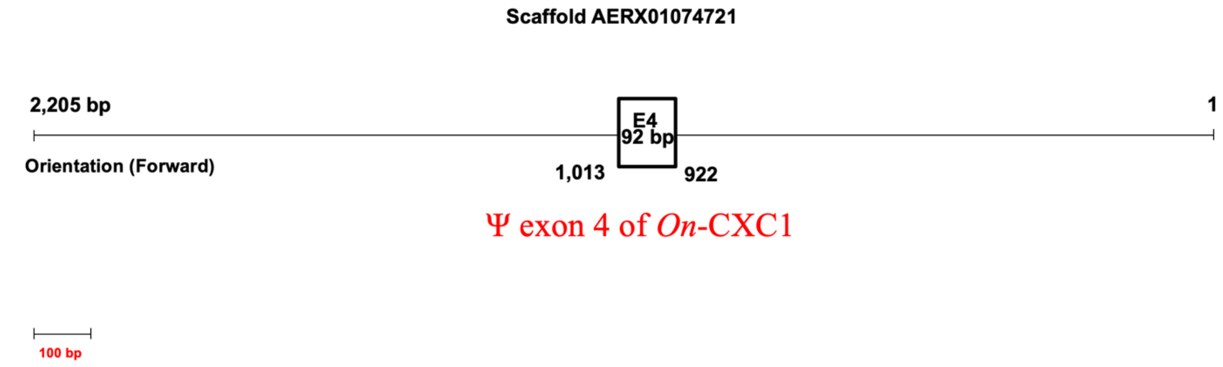

Figure S6A. Nakharuthai and Srisapoome (2020)

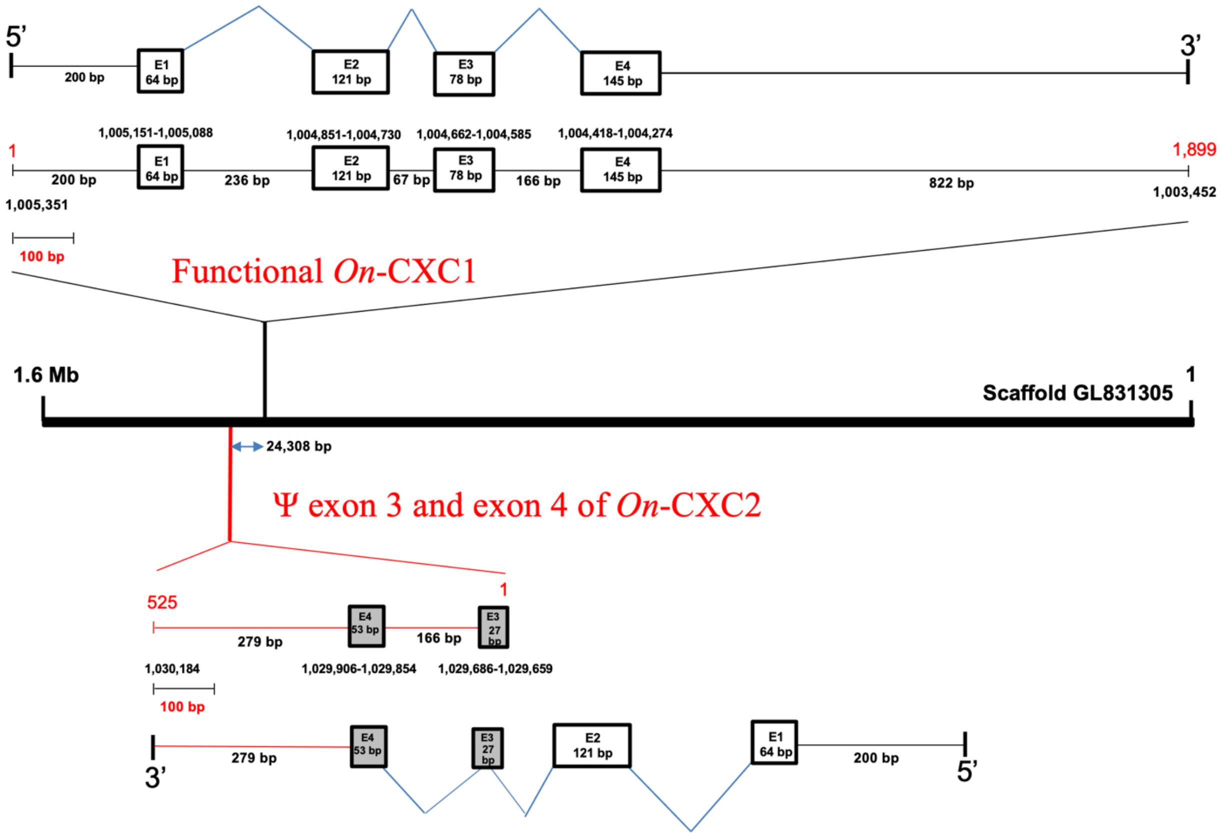

Figure S6B. Nakharuthai and Srisapoome (2020)

Supplementary Materials

Table S1. Oligonucleotide primers used in this study

Table S2. Homological analyses of nucleotide and amino acid sequences of *On*-CXC1 and *On*-CXC2 against CXC chemokines of other vertebrates.

Figure S1. Nucleotide and deduced amino acid sequences of the Nile tilapia CXC chemokine (*On*-CXC1) cDNA represented by the upper and lower lines, respectively. The start (ATG) and stop (TAG) codons are in bold. The signal peptide cleavage sites are underlined. Four cysteine residues are circled. Polyadenylation sites (ACTAAA, TATAAA and AATAAA) are underlined with a double line, and the 4 instability motifs (ATTTA) are underlined. The shaded letters indicate the $(GAA)_{12}$ microsatellite sequences.

Figure S2. Nucleotide and deduced amino acid sequences of the Nile tilapia CXC chemokine (*On*-CXC2) cDNA represented by the upper and lower lines, respectively. The start (ATG) and stop (TAG) codons are in bold. The signal peptide cleavage sites are underlined. Four cysteine residues are circled. Polyadenylation sites (AAAAAG, AAAACA, AATAGA and AATAAA) are underlined with a double line, and two instability motifs (ATTTA and ATTTTA) are underlined. The shaded letters indicate the $(GAA)_5$ microsatellite sequences.

Figure S3. Sequence alignment between *On*-CXC1 and *On*-CXC2. The four conserved cysteines (C) residues are boxed. The comparisons of the nucleotide and amino acid sequences of *On*-CXC1 and *On*-CXC2 are shown in the table.

Figure S4. Multiple sequence alignment of *On*-CXC1and *On*-CXC2 with other known CXC chemokines from various species. The four conserved cysteine residues are boxed and marked with asterisks. Gaps in the alignment are represented with dashes (-), and the ELR motif is shaded.

Figure S5. Phylogenetic tree showing the relationships between the Nile tilapia CXC chemokine (*On*-CXC1, *On*-CXC2) amino acid sequences and other known CXC chemokines from various species. The numbers at the relevant branches refer to bootstrap values (1,000 replicates).

Figure S6. Genomic structure and organization comparison of the Nile tilapia CXC chemokine genes with other known CXC chemokines of other vertebrates. (A) Scaffold AERX01074721 and (B) scaffold GL831305. The four exons are indicated by rectangles and the three introns by lines. The sizes of exons are indicated in the rectangles.
